# Small molecules to perform big roles: The search for Parkinson's and Huntington's disease therapeutics

**DOI:** 10.3389/fnins.2022.1084493

**Published:** 2023-01-09

**Authors:** Rodrigo Pérez-Arancibia, Marisol Cisternas-Olmedo, Denisse Sepúlveda, Paulina Troncoso-Escudero, Rene L. Vidal

**Affiliations:** ^1^Center for Integrative Biology, Faculty of Sciences, Universidad Mayor, Santiago, Chile; ^2^Departamento de Ciencias Básicas, Faculty of Medicine and Science, Universidad San Sebastián, Santiago, Chile; ^3^Biomedical Neuroscience Institute, Faculty of Medicine, University of Chile, Santiago, Chile; ^4^Center for Geroscience, Brain Health and Metabolism, Santiago, Chile; ^5^Molecular Diagnostic and Biomarkers Laboratory, Department of Pathology, Faculty of Medicine Clínica Alemana, Universidad del Desarrollo, Santiago, Chile

**Keywords:** Parkinson's disease, Huntington's disease, drug repurposing, pharmacological therapy, small molecules, natural products

## Abstract

Neurological motor disorders (NMDs) such as Parkinson's disease and Huntington's disease are characterized by the accumulation and aggregation of misfolded proteins that trigger cell death of specific neuronal populations in the central nervous system. Differential neuronal loss initiates the impaired motor control and cognitive function in the affected patients. Although major advances have been carried out to understand the molecular basis of these diseases, to date there are no treatments that can prevent, cure, or significantly delay the progression of the disease. In this context, strategies such as gene editing, cellular therapy, among others, have gained attention as they effectively reduce the load of toxic protein aggregates in different models of neurodegeneration. Nevertheless, these strategies are expensive and difficult to deliver into the patients' nervous system. Thus, small molecules and natural products that reduce protein aggregation levels are highly sought after. Numerous drug discovery efforts have analyzed large libraries of synthetic compounds for the treatment of different NMDs, with a few candidates reaching clinical trials. Moreover, the recognition of new druggable targets for NMDs has allowed the discovery of new small molecules that have demonstrated their efficacy in pre-clinical studies. It is also important to recognize the contribution of natural products to the discovery of new candidates that can prevent or cure NMDs. Additionally, the repurposing of drugs for the treatment of NMDs has gained huge attention as they have already been through clinical trials confirming their safety in humans, which can accelerate the development of new treatment. In this review, we will focus on the new advances in the discovery of small molecules for the treatment of Parkinson's and Huntington's disease. We will begin by discussing the available pharmacological treatments to modulate the progression of neurodegeneration and to alleviate the motor symptoms in these diseases. Then, we will analyze those small molecules that have reached or are currently under clinical trials, including natural products and repurposed drugs.

## 1. Introduction

As life expectancy has increased substantially during the past decades, the global prevalence of neurodegenerative disease has grown rapidly, as aging is one of the main factors involved in neurodegeneration (Hou et al., 2019; Azam et al., 2021). Among the wide range of neurodegenerative diseases, neurological motor disorders (NMDs) such as Parkinson's disease (PD) and Huntington's disease (HD) have increased their prevalence, having a negative impact on public health as the associated financial burden will continue to increase (Ou et al., 2021). Moreover, NMDs and other neurodegenerative diseases are not only a freight for the patients and public health systems, but also negatively impact the quality of life of caregivers, which are usually patient's family members (Achenbach and Saft, 2021; Rosqvist et al., 2022).

Even though patients from each NMD develop specific combination of motor symptoms, there are some common pathophysiological mechanisms in the development and progression of these diseases. The motor symptoms described in PD are triggered by the neurodegeneration of dopaminergic neurons, partly due to an accumulation of intraneuronal abnormal alpha-synuclein (α-syn) protein aggregates (Jankovic and Tan, 2020). The loss of dopaminergic neurons causes the decrease of an essential neurotransmitter: dopamine. Dopamine is produced in different areas of the nervous system, especially in the Substantia Nigra Pars Compacta (SNpc), and it is involved in multiple brain functions, such as behavior, cognition, motor activity, motivation, and reward (Schapira et al., 2017; Jankovic and Tan, 2020). Dopamine deficiency leads to a dysfunction of motor coordination in PD patients, among other problems.

On the other hand, Huntington's disease (HD) is an autosomal–dominant inherited neurological disorder caused by an unstable trinucleotide CAG repeat expansion at the N-terminus of the IT-15 gene, encoding the ~350 kDa huntingtin protein (Htt) (Jimenez-Sanchez et al., 2017; Tabrizi et al., 2020). This mutation causes Htt to contain an abnormally long polyglutamine (polyQ) tract, which confers Htt toxic properties (Tabrizi et al., 2020). HD prevalence was estimated at 2.7 per 100,000 inhabitants, with differences between regions of the world (McColgan and Tabrizi, 2018). A higher prevalence rate of HD has been reported in populations of Caucasian origin, not registering a difference between men and women (McColgan and Tabrizi, 2018). The clinical manifestation of HD is characterized by the loss of motor functions in the initial stages of the disease. Motor dysfunction continues throughout the progression of the disease, to what is added the appearance of psychiatric disorders and dementia (Tabrizi et al., 2020). Patients follow a relentless course of cognitive and motor impairment, which ultimately leads to death between 12 and 15 years from the onset of the symptoms (Tabrizi et al., 2020). Recent evidence indicates that mutant huntingtin (mHtt) aggregation in susceptible neurons may be responsible for the onset and progression of HD phenotypes, and death of affected neurons are associated with the accumulation of mutant proteins in insoluble aggregates (Tabrizi et al., 2020).

Despite numerous efforts being made to better understand the cellular mechanisms underlying the development of these disorders, to date there are no treatments that can prevent, cure, or significantly delay the progression of the disease (Troncoso-Escudero et al., 2020). Moreover, the current pharmacological strategies are only palliative and do not slow down or reverse the progression of the disease. As an example, pharmacological approaches for PD treatment aim to correct the loss of the fine motor control using drugs that ***(1)*** increase dopamine availability with dopamine precursors (**Levodopa**) or dopaminergic agonists (**Pramipexole**), and that ***(2)*** inhibit dopamine degradation by the monoamine-oxidase (**Selegeline** and **Rasagiline**) or catechol-o-metyltransferase (**Entocapone** and **Tolcapone**) (Van der Schyf, 2015; Carrera and Cacabelos, 2019). The chronic administration of antiparkinsonian drugs results in a “*wearing-off phenomenon*,” which produces additional psychomotor and autonomic complications, like dyskinesia (Ammal Kaidery et al., 2013; Cacabelos, 2017). Moreover, L-dopa pharmacokinetics are unpredictable and commonly lead to increased administration, complex regimens, and poor patient compliance. Nevertheless, L-dopa treatment remains as the gold standard treatment for movement disorder in PD patients (Oertel and Schulz, 2016; Fox et al., 2018).

For HD, only one approved pharmacological therapy for the treatment of motor symptoms has been approved: **tetrabenazine** (Yero and Rey, 2008). This is a small molecule that reduces dopamine neurotransmission *via* its depletion from presynaptic vesicles by inhibition of the vesicular monoamine transporter 2 (VMAT-2) (Yero and Rey, 2008). **Tetrabenazine** treatment results in a reduction of the chorea symptom in HD patients (Rodrigues and Wild, 2020). Nevertheless, sedation, anxiety, depression, and suicidality, have been observed as side effects of its administrations, and its use is limited in HD patients (Group et al., 2016; Rodrigues and Wild, 2020). On 2017, the FDA approved a deuterated derivative of tetrabenazine, deutetrabenazine, which has an improved pharmacokinetic profile that enables a less frequent daily dosage with comparable systemic exposure of the drug. The improved pharmacokinetics results in a better safety profile of the drug (Group et al., 2016).

Given that the current FDA-approved drugs for the treatment of PD and HD have several drawbacks, different therapeutic alternatives have been explored for the development of new treatments. In this context, novel therapeutic strategies such as cellular replacement therapies and gene editing, among others, have gained attention as they have shown to effectively reduce the load of toxic protein aggregates in different models of neurodegeneration, and thus, hold the potential to have disease modifying effects (Troncoso-Escudero et al., 2020). Cellular replacement therapies were among the first treatments used for PD and HD, which looked to restore the neuronal populations lost in the brain of the affected patients. Despite some functional recovery being observed in treated patients, important religious and ethical concerns regarding the use of fetuses arose (Troncoso-Escudero et al., 2020). Because of this, strategies to obtain dopaminergic or GABAergic neurons from embryonic stem cells and induced pluripotent stem cells emerged, with promising results in animal models of PD and HD (Troncoso-Escudero et al., 2020). Interestingly, an ongoing clinical trial will evaluate if dopamine progenitor cells derived from pluripotent stem cells injected into the putamen of 10 PD patients is safe and well tolerable, and if patients present potential side effects (**NCT: 04802733**). In addition to cell replacement, other strategies have looked to correct the mutated genes found in PD and HD patients. Several gene silencing/editing technologies have been applied for this, such as RNA interference, antisense oligonucleotides and CRISPR/cas9. Many clinical trials using gene therapy for PD have been completed, showing modest improvements in motor function (Troncoso-Escudero et al., 2020). Two promising clinical trials were evaluating the use of antisense oligonucleotides to reduce the levels of HTT (**IONIS-HTTRx; NCT03342053, NCT03761849**) and mHTT **(PRECISION HD1/2; NCT03225833, NCT03225846**) in the brains of HD patients. Unfortunately, both studies have been terminated due to lack of efficacy of the treatments.

Considering all the above, the lack of consistent and clinically relevant results for gene and cellular therapy, together with the cost and difficulties for delivery, highlights the urgent need to investigate other therapeutic approaches. In this critical context and considering the numerous efforts that have helped to define and/or re-define the pathophysiological mechanisms underlying NMDs, there is an interesting avenue for the rational design and development of new small molecules that target those mechanisms to halt and/or revert the progression of NMDs. Moreover, we can also explore the chemical space of natural products to discover new therapies for NMDs, an strategy that is supported by several epidemiological studies (Sanadgol et al., 2017; Polito et al., 2018; Rahman et al., 2021; Uddin et al., 2021) and the ceaseless relevance of natural products as drug leads (Newman and Cragg, 2020). Furthermore, “old drugs” can still be useful in drug discovery efforts through drug repurposing, as these compounds have already been proved safe and thus, the development costs would be potentially lower, and the development timelines should be shorter (Pushpakom et al., 2019). In this review, we discuss recent advances in the discovery of new small molecules, natural products or natural extracts, and drug repurposing for the treatment NMDs. Given the extensive amount of literature regarding small molecules under investigation for the treatment of NMDs (Elkouzi et al., 2019; Dash et al., 2021; Devadiga and Bharate, 2022; Liu et al., 2022), this review focuses primarily on those strategies that have reached clinical trials and thus, have potential to reach approval for the treatment of NMDs.

## 2. Synthetic small molecules for PD and HD

**Figure 1:**
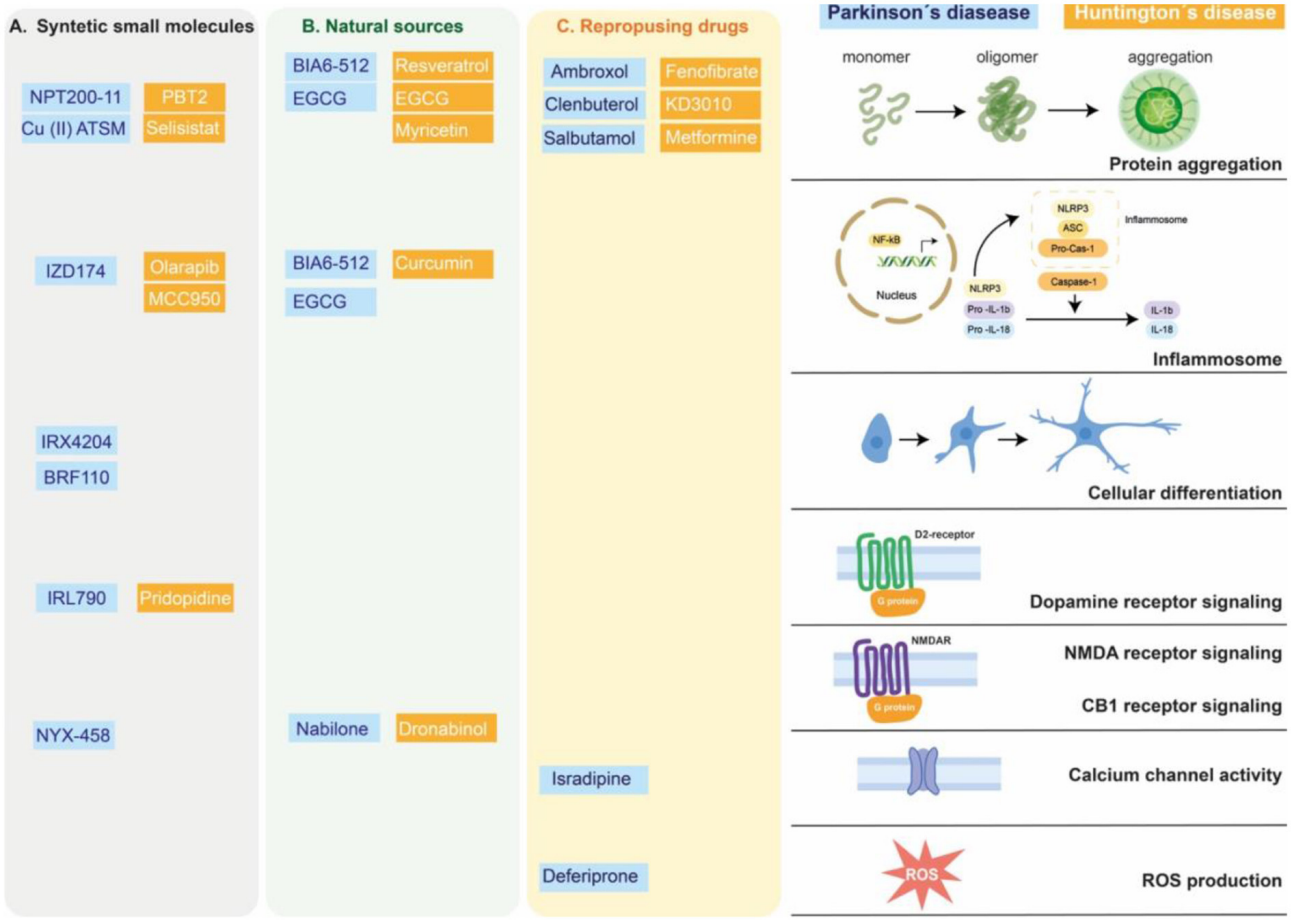
Targets of small molecules in neurodegenerative motor disorders. Synthetic small molecules **(A)**, small molecules from natural sources **(B)** or Repurposing FDA-approved drugs **(C)** used to alleviate clinical symptoms of Parkinson's disease (blue box) or Huntington's disease (orange box). These molecules participate in modified specific cellular pathways related to alleviating or delaying the neurodegenerative process, as protein aggregation, inflammatory response (inflammosome), cellular differentiation, G protein coupled receptor, calcium channel activity or reactive oxygen species (ROS) production.

In this section, we will focus on those synthetic small molecules that have reached clinical trials for the treatment of PD and HD. Nevertheless, we also considered synthetic small molecules that are still under pre-clinical investigation given that they represent novel and promising strategies for the treatment of PD and HD (Figure 1A).

### 2.1. Synthetic small molecules for PD treatment

#### 2.1.1. Synthetic small molecules that modulate protein aggregation levels

Although several strategies have been explored for PD treatment (Troncoso-Escudero et al., 2020), the identification of α-syn accumulation as one of the key pathological features of this disease has led to the development of pharmacological strategies that directly or indirectly target α-syn misfolded to prevent its aggregation and all the toxic events associated to aggregate formation (Savitt and Jankovic, 2019; Teil et al., 2020). One example of small molecules that directly modulates protein aggregation is **NPT200-11**, a totally synthetic small molecule derivative of **NPT100-18A**, a *de novo* synthetized molecule modeled to target specific regions of the α-syn protein that are key players in oligomer formation (Wrasidlo et al., 2016). Although treatment with **NPT100-18A** showed the effect of decreasing the α-syn accumulation *in vitro* and *in vivo* models of PD (Wrasidlo et al., 2016), its poor bioavailability led to the later development of **NPT200-11**, which retained its effects over α-syn aggregation with improved physicochemical and pharmacokinetic properties (Price et al., 2018). Recently, oral treatment with **NPT200-11** showed the ability to reach the brain and to exert positive effects over neuropathological and behavioral endpoints related to its ability to reduce α-syn aggregation in a transgenic mouse model of PD (Price et al., 2018). The safety and tolerability of **NPT200-11** have already been analyzed in a clinical trial where the drug was administered to healthy subjects (NCT02606682, Table 1), but its safety and efficacy in PD patients remains to be elucidated. Interestingly, **UCB0599**, the *R*-enantiomer of NPT200-11, has shown an acceptable safety and tolerability profile (Smit et al., 2022) and is currently under a phase 2 clinical trial to determine its effects over clinical symptoms of PD (NCT04658186, Table 1).

**Table 1:** Description of synthetic small molecules used in Parkinson's disease or Huntington's disease.

			**Clinical trials**							
**Name**	**Target**	**Preclinical models (references)**	**CT identifier**	**Phase**	**First posted**	**Primary purpose**	**Population**	**Study design**	**Primary outcomes**	**Results**
**NPT200-11**	Specific regions of the α-syn protein	PD mice models (Rahman et al., 2021)	NCT02606682	Phase 1	2015	Treatment	Healthy subjects	55 participants	Safety, as determined by the number of participants with adverse events related to treatment, the number of participants with clinically significant changes in blood pressure, heart rate and respiration, the number of participants with abnormal laboratory values, the number of participants with abnormal ECGs. [Time frame: screening (28 days prior to dosing) through Day 7]	No study results posted
**UCB0599**	R-enantiomer of NPT200-11		NCT04658186	Phase 2	2020	Treatment	PD patients	450 participants	Movement disorder society unified Parkinson's disease rating scale (MDS-UPDRS) Parts I-III sum score [Time frame: from baseline up to 18 months]	No study results posted
**MCC950**	NLRP3 inhibitors	PD mice models (Sanadgol et al., 2017)								
**IZD174**	NLRP3 inhibitors		NCT04338997	Phase 1	2020	Basic science	PD patients	0 participants	Concentration of IZD174 in plasma [time frame: pre-dose to 36 hours post dose]. Plasma PK profile following an intra-individual dose escalation of IZD174	No study results posted
**BRF110**	Activate the NURR1-RXR heterodimer	PD mice models (Elkouzi et al., 2019)								
**IRX4204**	Activate the NURR1-RXR heterodimer	PD rats models (Dash et al., 2021)	NCT02438215	Phase 1	2015	Treatment	Early stage PD	15 participants	The percent change from baseline to end of dosing period (Day 30) of the striatal binding ratio (SBR). [Time frame: 30 days]	No study results posted
**Cu (II) ATSM**	Inhibit the peroxynitrate-induced α-syn aggregation	PD mice models (Wrasidlo et al., 2016)	NCT03204929	Phase 1	2017	Treatment	Early idiopathic Parkinson's Disease	31 participants	Recommended phase 2 dose as determined by the number of patients in each dose cohort with intolerance over up to 6 months treatment. [Time frame: 6 months]	No study results posted
**IRL790**	Dopamine antagonist, antagonize the D3/D2 receptors	PD rats models (Smit et al., 2022)	NCT03368170	Phase 2	2017	Treatment	PD patients	75 participants	The change from baseline to day 28 of treatment (Visit 4) in the sum of the items comprising the Unified Dyskinesia Rating Scale (UDysRS). The Unified Dyskinesia Rating Scale (UDysRS) is administered to assess dyskinesia. The scoring range is 0–104, where higher score means more dyskinesia. [Time frame: baseline and 4 weeks]	Analyze its safety and tolerability in PD patients and might have a positive impact on the quality of life of PD patients suffering of dyskinesia and psychosis
**NYX-458**	Modulator of the NMDA receptor	PD primates models (Wang et al., 2019)	NCT04148391	Phase 2	2019	Treatment	PD patients with mild cognitive impairment	99 participants	Change from baseline in physical examination [Time frame: subjects will be followed up to 14 days post-dose]	No study results posted
**PBT2**	Metal chelator	HD mice models (Kelley et al., 2019)	NCT01590888	Phase 2	2012	Treatment	Early to mid-stage HD	109 participants	Safety and tolerability of PBT2 in patients with HD. As measured by the total number of participants in each dose group who reported at least one adverse events during the study, [Time frame: baseline to 26 weeks]	Treatment with PBT2 also showed to improve the cognitive performance of treated patients, but a larger study is needed in order to confirm its beneficial effects in HD patients
**Selisistat**	Inhibitor of the Sirtuin 1 deacetylase (SirT1)	HD mice models (Decressac et al., 2013)	NCT01485965; NCT01485952; NCT01521585; NCT01521832	Phase 1; Phase 1; Phase 2; Phase 1	2011; 2011; 2012; 2012	Treatment	HD patients	26 participants; 55 participants; 144 participants; 88 participants	To determine the effect of food on the repeated dose pharmacokinetics of SEN0014196 at 100 mg once daily in subjects with Huntington's disease [Time frame: 14 days]; To determine the change from baseline of a series of pharmacodynamic markers in peripheral blood mononuclear cells [Time frame: baseline, day 7, day 14, follow-up]; Safety and tolerability. Adverse event (AE) reporting, 12-lead electrocardiogram (ECG), vital signs, physical examination findings, and laboratory safety tests. Suicide risk (Columbia Suicide Severity Rating Scale, C-SSRS) [Time frame: 12 weeks]; Safety and tolerability of ascending single and multiple oral doses of SEN0014196 in healthy male and female subjects. Vital signs, cardiovascular and neurological function, laboratory safety parameters. Type and frequency of adverse events[Time frame: up to 7 days after single dose and up to 10 days following multiple dose]	No study results posted
**Pridopidine**	Dopamine D2 receptor ligand	HD mice models (Khan et al., 2018; Waters et al., 2020)	NCT03019289	Phase 1	2017	Other	Healthy subjects and HD patients	23 participants	Sigma-1 receptor occupancy. Receptor occupancy of pridopidine to Sigma-1 receptors (S1R) in the brain was assessed from Positron Emission Tomography (PET) imaging with (S)-(-)-[18F]fluspidine [Time frame: 2 hours after oral administration of pridopidine]	Safety, tolerability and efficacy as already been tested, but It's properties as a disease modifying treatment for HD remain to be analyzed
**Pridopidine**	Dopamine D2 receptor ligand	HD mice models (Khan et al., 2018; Waters et al., 2020)	NCT01306929; NCT02006472	Phase 2	2011; 2013	Treatment	HD patients	134 participants; 408 participants	Number of patients with at least one adverse event [Time frame: from signing of the informed consent through the end of the follow-up period, which was defined as 30 days after the final study visit in an individual patient, an average of 2.8 years]; Change From Baseline in Unified Huntington's Disease Rating Scale-Total Motor Score (UHDRS-TMS) at Week 26. TMS was defined as the sum of all UHDRS motor domains ratings. The motor section of the UHDRS assesses motor features of Huntington's Disease (HD) with standardized ratings of oculo-motor function, dysarthria, chorea, dystonia, gait, and postural stability. Each of 15 assessments is rated on a scale of 0 (normal) to 4 (marked impairment) for a TMS range of 0–124. Negative change from baseline values indicate improvement [Time frame: 26 weeks].	Safety, tolerability and efficacy as a symptomatic treatment of this drug has already been tested, but it's properties as a disease modifying treatment for HD remain to be analyzed
**Pridopidine**	Dopamine D2 receptor ligand	HD mice models (Khan et al., 2018; Waters et al., 2020)	NCT00724048; NCT00665223	Phase 3	2008	Treatment	HD patients	227 participants; 437 participants	Sum score of items 4–10 and 13–15 of the UHDRS motor assessment. The primary objective is to assess the effects of ACR16 on voluntary motor function in HD patients, as defined as the sum score of items 4–10 and 13–15 of the UHDRS motor assessment (a modified motor score in MS) at 26 weeks of treatment [Time frame: 26 weeks]; The primary objective is to assess the effects of ACR16 on voluntary motor function in HD patients, as defined as the sum score of items 4–10 and 13–15 of the UHDRS motor assessment (a modified motor score mMS) at 26 weeks of treatment [Time frame: last timepoint at which outcome is assessed is after 26 weeks]	Safety, tolerability and efficacy as a symptomatic treatment of this drug has already been tested, but it's properties as a disease modifying treatment for HD remain to be analyzed

#### 2.1.2. Synthetic small molecules modulating other cellular pathways

##### 2.1.2.1. NLRP3 inhibitors

The recognition of the role in the loss of dopaminergic neurons of the activation of the NLR family pyrin domain containing 3 (NLRP3) inflammasome by α-syn aggregates in PD pathology accounts for another pharmacological target that can be exploited for PD treatment (Gordon et al., 2018; Wang et al., 2019; Haque et al., 2020). The NLRP3 inflammasome is a key component of the innate immune system involved in the activation of caspase-1 and the secretion of pro-inflammatory cytokines such as IL-1beta and IL-18 (Kelley et al., 2019) and, thus, it plays an important role in the pathogenesis of inflammation-related diseases. Also, extracellular α-syn acts as a pro-inflammatory molecule, which can trigger the activation of NLRP3, contributing to the inflammatory state in the PD brain (Otani and Shichita, 2020). In a proof-of-concept experiment, the oral treatment with NLRP3 inhibitor **MCC950** was able protect dopaminergic neurons from α-syn aggregates-induced activation of the inflammasome, improving the motor performance in a preclinical mouse model of PD, but due to patent problems **MCC950** cannot be commercialized (Gordon et al., 2018). **IZD174** is a small molecule inhibitor of NLRP3 that will be part of a phase 1 clinical trial with PD patients that is currently recruiting subjects (**NCT04338997**, Table 1) and which will evaluate its pharmacokinetics and pharmacodynamics after oral treatment. Although targeting the NLRP3 inflammasome seems like a promising strategy for PD treatment, the lack of information on how the activation of the NLRP3 inflammasome can directly affect the survival of dopaminergic neurons has slowed down the progress in this direction (Haque et al., 2020).

##### 2.1.2.2. NURR1 activators

Another target with pharmacological potential for the treatment of PD is the NURR1 transcription factor. This transcription factor is involved in the development and differentiation of dopaminergic neurons in the midbrain and has an important function in the continuing maintenance of these types of cells under both physiological conditions and stress (Decressac et al., 2013). NURR1 can form heterodimers with the retinoid X receptor (RXR) and, consequently, RXR ligands can modulate the functions of NURR1 (Wang et al., 2016). **BRF110** is a synthetic small molecule designed to specifically activate the NURR1-RXR heterodimer, whose oral treatment has shown to protect dopaminergic neurons from α-syn and to restore dopamine synthesis in preclinical models of PD (Spathis et al., 2017). Nevertheless, to our knowledge, no clinical trials related to **BRF110** have been posted to date**. IRX4204** is another synthetic RXR ligand that can cross the BBB to specifically activate the NURR1-RXR heterodimer (Wang et al., 2016). The oral treatment with this drug significatively reduced PD motor symptoms, restored the dopaminergic neuron loss, and increased the expression of molecules related to dopamine synthesis (Wang et al., 2016). A phase 1 clinical trial of **IRX4204** in patients with early-stage PD has already been completed (NCT02438215, Table 1), showed that the drug was safe and well tolerated, but no changes in dopamine transporter binding was observed (Sanders et al., 2016). Interestingly, patients presented a trend toward improvement in motor symptoms, opening the window for further analysis of IRX4204 (Sanders et al., 2016).

#### 2.1.3. Other synthetic small molecules for PD treatment

**Cu (II) ATSM** is a cupper-containing radiolabeled imaging agent that has shown to inhibit α-syn aggregation *in vitro*, which mediated cell toxicity, and dopaminergic neuron loss in preclinical models of PD (Hung et al., 2012). In a study aiming to analyze other pathways involved in the neuroprotective activities of **Cu (II) ATSM**, a RNAseq whole transcriptome sequencing approach was utilized to evaluate the changes in the SNpc upon treatment with **Cu (II) ATSM** in a mouse model of PD (Cheng et al., 2016). Results showed a set of genes related to brain and cognitive development, neuroplasticity, regulation and cellular response, whose expression was recovered upon oral treatment with **Cu (II) ATSM** in PD animals (Cheng et al., 2016), thus the beneficial effects of **Cu (II) ATSM** treatment are not only related to its indirect effects over protein aggregation but might also be related to its ability to restore gene expression. The safety and tolerability of **Cu (II) ATSM** treatment in PD patients has recently been analyzed in a multicenter phase 1 clinical trial (NCT03204929, Table 1), although results haven't been posted yet.

Although it was not developed as a disease-modifying treatment, **IRL790 (Mesdopetam)** is a novel compound that has already been tested in clinical trials to analyze its safety and tolerability in PD patients (Svenningsson et al., 2018) and, recently, its efficacy in the treatment of PD-related dyskinesia has also been evaluated (NCT03368170, Table 1). **IRL790** is a dopamine antagonist that was rationally developed to antagonize the D3/D2 receptors (Waters et al., 2020). Upon oral treatment, **IRL790** was able to exert anti-dyskinesic and anti-psychotic effects in preclinical models without having a negative impact on the normal dopamine transmission and, in consequence, in the normal motor function (Waters et al., 2020). Hence, **IRL790** treatment might have a positive impact on the quality of life of PD patients suffering from dyskinesia and psychosis.

**NYX-458** is another example of a non-disease-modifier drug being developed for the treatment of PD. Specifically, **NYX-458** is a modulator of the NMDA receptor that has shown the effect of facilitating synaptic plasticity (Khan et al., 2018) and chronic low-dose oral treatment with this drug resulted in an improvement of the cognitive performance in a primate model of PD (Barth et al., 2020). The safety and tolerability of **NYX-458** is being tested in a phase 2 clinical trial on PD patients with mild cognitive impairment, where the drug's effects on cognitive performance are also being explored (NCT04148391, Table 1). This clinical trial is expected to be completed by the end of 2022.

### 2.2. Synthetic small molecules for HD treatment

#### 2.2.1. Synthetic small molecules that modulate protein aggregation levels

Strategies that directly or indirectly target mHTT aggregation have also been under development for HD treatment (Tabrizi et al., 2019). As in PD, the rationale behind targeting mHTT aggregation is in line with its pathogenic role in the progression of the disease. **PBT2** is an orally available metal chelator with the ability to prevent protein aggregation associated with metal-mediated oxidative stress (Cherny et al., 2012). The oral treatment with **PBT2** has shown to reduce neuronal damage, reverse motor dysfunction and extend lifespan in preclinical models of HD which express polyQ extended proteins that are able to form toxic aggregates (Cherny et al., 2012). The safety, tolerability, and efficacy of **PBT2** has already been tested in a phase 2 clinical trial including patients with early to mid-stage HD (NCT01590888, Table 1). The results of this clinical trial showed that oral treatment with **PBT2** is generally well tolerated and safe, as the incidence of serious adverse effects in all treated patients was low (Huntington Study Group Reach2HD Investigators, 2015). Furthermore, treatment with **PBT2** also showed to improve the cognitive performance of treated patients, but a larger study is needed in order to confirm its beneficial effects in HD patients (Huntington Study Group Reach2HD Investigators, 2015).

**Selisistat** is a small molecule inhibitor of the Sirtuin 1 deacetylase (SirT1), whose use as treatment has shown to protect against mHTT toxicity in cellular and preclinical models of HD (Smith et al., 2014). Its significant effects over mHTT aggregation produced an improvement in the motor function and lifespan of treated animals (Smith et al., 2014). The effects of **selisistat** and other SirT1 inhibitors over protein aggregation can be explained by the requirement of post-translational acetylation for the clearance of mHTT through the autophagy machinery (Jeong et al., 2009). Four clinical trials of **selisistat** treatment in HD patients have already been performed (NCT01485965; NCT01485952; NCT01521585; NCT01521832, Table 1), from where its safety and tolerability have been confirmed (Süssmuth et al., 2015). Although **selisistat** treatment did not show adverse effects on the motor and cognitive performance of HD patients (Süssmuth et al., 2015), its efficacy as a disease-modifying treatment of HD remains to be evaluated.

#### 2.2.2. Synthetic small molecules modulating other pathways

As previously explained, the activation of NLRP3 inflammasome by α-syn aggregates has been related to neuronal death in PD (Gordon et al., 2018; Wang et al., 2019; Haque et al., 2020). Recently, Paladino et al showed that the expression of NLRP3 was significantly higher in R6/2 mice compared to control mice (Paldino et al., 2020). Moreover, oral treatment of R6/2 animals with **Olarapib**, an FDA approved drug commonly used for the treatment of ovarian cancer, significantly reduced the expression of NLRP3, the levels of neuroinflammation, and promoted neuroprotection in treated animals (Paldino et al., 2020). More recently, Chen et al. (2022), showed that oral treatment with the highly selective NLRP3 inhibitor **MCC950** suppressed NLRP3 inflammasome, reduced levels of pro-inflammatory cytokines and neuronal toxicity in the R6/2 preclinical model and, more importantly, improved motor dysfunction and extended lifespan in treated animals compared to non-treated mice.

**Pridopidine** is a dopamine D2 receptor ligand that upon interaction destabilize the active conformation of the receptor and, consequently, acts as a dopamine antagonist increasing synthesis, release, and metabolism of dopamine in subcortical areas (Waters et al., 2018). Preclinical data shows that treatment with **pridopidine** has a higher affinity for the sigma 1 receptor, which is in the endoplasmic reticulum (ER), and upon interaction with pridopidine promotes neuroprotection in mouse primary cortical neurons and patient-derived induced pluripotent stem cells (Eddings et al., 2019). Furthermore, **pridopidine** treatment has also shown to improve behavioral symptoms and reverse the changes in gene transcription related to HD in a preclinical model of the disease (Garcia-Miralles et al., 2017; Kusko et al., 2018). The safety, tolerability, and efficacy as a symptomatic treatment of this drug has already been tested in phase 1 (NCT03019289, Table 1), phase 2 (NCT02006472, NCT01306929, Table 1) and phase 3 (NCT00724048, NCT00665223, Table 1) clinical trials. These studies demonstrated that 90 mg of **pridopidine** acts as selective S1R ligand (Grachev et al., 2021) and that treatment of HD patients with pridopidine results in significant reduction in the decline in Total Functional Capacity, a scale used to assess independence, perform domestic work, among other tasks (McGarry et al., 2020a,b). Further studies are needed to determine its properties as a disease modifying treatment for HD.

## 3. Small molecules from natural sources for PD and HD drug discovery

Although it's been a long time since the golden era of natural products drug discovery, small molecules from natural sources still play an important role in drug development (Newman and Cragg, 2020). Among the most distributed molecules in the plant kingdom are polyphenolic compounds, with more than 10,000 different molecules described to date (Figueira et al., 2017). The consumption of phenolic-rich foods has been identified by several epidemiologic studies as beneficial for cognitive function (Commenges et al., 2000; Devore et al., 2010; Lefèvre-Arbogast et al., 2018), although there have been some controversies (Crichton et al., 2013; Reale et al., 2020). The accumulation of evidence has led to the preclinical analysis of the potential role of polyphenolic compounds in the prevention and treatment of neurodegenerative diseases by different studies (revised in Maher, 2019). In this section we will focus on *in vivo* and clinical evidence for the use of polyphenolic compounds and polyphenolic-rich plant extracts for prevention or treatment of PD and HD. The neuroprotective properties of other types of phytochemicals and plant extracts can be found in Figure 1B (Choudhary et al., 2013; Zhang et al., 2017; Javed et al., 2018).

### 3.1. Polyphenolic compounds in PD

#### 3.1.1. Resveratrol

Previous reviews have analyzed the potential neuroprotective capacity of polyphenolic compounds, showing that these types of compounds could be acting through their antioxidant, anti-inflammatory, and anti-aggregation activities (Zhang et al., 2017; Javed et al., 2018; Kujawska and Jodynis-Liebert, 2018; Maher, 2019). One of the most studied polyphenolic compounds is **resveratrol**. **Resveratrol** is a stilbene most notably found in grapes and blueberries, whose antioxidant and anti-inflammatory activities have been extensively studied (revised in Diaz-Gerevini et al., 2016; Repossi et al., 2020). Its neuroprotective properties have also been analyzed and several mechanisms of action of this molecule have been described (Bastianetto et al., 2015; Kou and Chen, 2017; Gomes et al., 2018; Lin et al., 2018). In different *in vivo* PD models, **resveratrol** has shown the ability to delay the progression of the disease and improve motor function through the rescue of dopaminergic neurons (Huang et al., 2019), induction of α-syn degradation by autophagy (Guo et al., 2016), inhibition of α-syn oligomerization and reduction of neuroinflammation and oxidative stress (Zhang et al., 2018). Also, the concomitant use of **resveratrol** and **L-dopa** in a mouse model of PD has been effective in reducing the dopaminergic neuron loss and motor dysfunction, with the advantage of a lower administration of L-dopa, and therefore, less side effects manifestation (Liu et al., 2019). The potential applications of **resveratrol** in the treatment of PD and other diseases are limited by its poor solubility and extensive hepatic metabolisms which lowers its bioavailability (Salehi et al., 2018; Arbo et al., 2020). This issue has led to the development of **resveratrol derivatives** with improved pharmacokinetics and similar biological effects, whose potential uses in PD treatment are reviewed in (Arbo et al., 2020). **BIA6-512** is a resveratrol derivative (**trans-resveratrol**) whose pharmacokinetics have been analyzed in clinical trials as monotherapy (NCT03095105 and NCT03093389, Table 2) and in combination with **L-dopa** (NCT03091543, Table 2), but no clinical trials studying the efficacy of **BIA6-512** treatment, alone or in combination, have been performed to date.

**Table 2:** Description of small molecules from natural sources used in Parkinson's disease or Huntington's disease.

					**Clinical trials**							
**Name**	**Composition**	**Source plants**	**Target**	**Preclinical models (references)**	**CT identifier**	**Phase**	**First posted**	**Primary purpose**	**Population**	**Study design**	**Primary outcomes**	**Results**
**Resveratrol**	Polyphenolic compounds	Grapes and blueberries	Anti-oxidant, anti-inflammatory, and anti-aggregation. Activation of the ERK signaling pathway	PD mice models (Choudhary et al., 2013; Crichton et al., 2013; Maher, 2019; Reale et al., 2020); HD mice models (Pinto et al., 2015; Buhmann et al., 2017, 2019)	NCT02336633	Not Applicable	2015	Treatment	HD patients	102 participants	Rate of caudate atrophy, Measurement of the rate of caudate atrophy before and after 1 year of treatment with resveratrol in early affected HD patients using volumetric MRI [Time frame: 1 year]	No study results posted
**BIA6-512**	Resveratrol derivative		Anti-oxidant, anti-inflammatory, and anti-aggregation		NCT03095105; NCT03093389; NCT03091543	Phase 1	2017	Treatment	PD patients	25 participants; 40 participants; 20 participants	Cmax—the maximum observed plasma concentration (single dose) [Time Frame: Day 1: pre-dose 1, and $\frac{1}{4}$, $\frac{1}{2}$, $\frac{3}{4}$, 1, 1$\frac{1}{2}$, 2, 3, 4, 6, 8, 12 and 16 h post-dose]; Cmax—the maximum plasma concentration—first dose (dose 1) [Time Frame: Dose 1: pre-dose and $\frac{1}{4}$, $\frac{1}{2}$, $\frac{3}{4}$, 1, 1$\frac{1}{2}$, 2, and 3 hours post-dose]. BIA 6-512 pharmacokinetic parameters following the first dose (Dose 1); Maximum observed plasma drug concentration (Cmax) post-dose—Levodopa [Time frame: pre-dose, $\frac{1}{4}$, $\frac{1}{2}$, 1, 1$\frac{1}{2}$, 2, 3, 4, 6, 9, 12, 16 and 24 hours post-dose]. Pharmacokinetic parameters of Levodopa following oral administration of single-doses of Madopar^®^ HBS 125 concomitantly with placebo or BIA 6-512 25, 50, 100, and 200 mg	No study results posted
**Green tea**	Polyphenolic compounds			PD mice models (Petrussa et al., 2013)	NCT00461942	Phase 2	2007	Treatment	PD patients	480 participants	Delay of progression of Motor dysfunction	No study results posted
**EGCG**	Flavonoid	Green tea	Anti-oxidant, anti-inflammatory, and anti-aggregation. Activation of the ERK signaling pathway	PD mice models (Liu et al., 2019); HD fly models (Cristino et al., 2020; Morales and Jagerovic, 2020)	NCT01357681	Phase 2	2011	Treatment	HD patents	54 participants	Change of cognitive functions (UHDRS-Cognition: composite score of Stroop test, Verbal fluency & Symbol Digit Modalities Test) after 12 months in comparison to baseline [Time frame: month 0, month 12]	Effects on cognitive function of EGCG treatment for 1 year in patients with HD
**Nabilone**	Phytocannabinoids	Cannabis sativa	Anti-oxidant and CB2 receptor agonist	PD mice and rats models (Nagle et al., 2006; Xu et al., 2016; Di Meo et al., 2020)	NCT03769896; NCT03773796	Phase 2; Phase 3	2018	Treatment	PD patients	48 participants; 22 participants	Changes of non-motor symptoms. changes in movement disorders society—unified Parkinson's disease rating scale (MDS-UPDRS) part I minimum points: 0, maximum points: 52, higher score values indicate a worse outcome [Time frame: from baseline to 4 weeks + 2 days]; AEs in PD patients taking Nabilone, between V 1 and V 3. Safety and tolerability will be evaluated with reference to the following: adverse events (AE). [Time frame: 6 months]	Changes in motor and different non-motor symptoms of PD
**Curcumin**	Polyphenolic compounds	Curcuma	Anti-oxidant, anti-inflammatory.	HD mice models (Peball et al., 2019)								
**Dronabinol**	Synthetic Δ^9^-THC				NCT01502046	Phase 2	2011	Treatment	HD patents	25 participants	Serious adverse events reported [Time frame: 8 months]. Changes in the UHDRs score [Time frame: On week 4 and 12 of each period], UHDRS scale scores from the following perspectives: motor, cognitive, psychiatric and functional	No study results posted

#### 3.1.2. Flavonoids

Flavonoids are one of the biggest family of polyphenolic compounds found in plants with around 5000 molecules described, classified by the degree of oxidation of their central ring, their hydroxylation pattern, and the presence of substitution in C3 (Petrussa et al., 2013; Maher, 2019). These compounds can be found in a variety of foods and food products such as grapes, berries, tea and cocoa (Terahara, 2015) and their neuroprotective effects have been extensively analyzed in preclinical models (revised in Vauzour et al., 2008; Spencer, 2009; Vauzour, 2014; Flanagan et al., 2018; Gildawie et al., 2018; Bakoyiannis et al., 2019; Maher, 2019; Di Meo et al., 2020). Nevertheless, our search for clinical trials of flavonoids related to PD revealed that not many of these plant metabolites reach the clinical stage for the treatment of neurodegenerative diseases. In this context, one clinical trial in phase 2 of **green tea polyphenols** was found. In this clinical trial performed in China (NCT00461942, Table 2), the safety and efficacy of the treatment with a green tea extract was analyzed in PD patients not yet taking prescribed PD-specific medications, but results are not posted. The rationale behind the use of a green tea extract is the presence of **catechins**, specifically **EGCG** (epigallocatechin-3-gallate), a polyphenolic compound of the **flavonoid** family (Nagle et al., 2006). **EGCG** treatment has shown the ability to inhibit α-syn in *in vitro* and human *postmortem* tissue models of PD by directly interacting with specific amino acid sequences in the protein and blocking aggregation-prone regions by intermolecular hydrophobic interactions (Xu et al., 2016). Furthermore, **EGCG** oral treatment rescued striatal dopaminergic neurons by protecting them against oxidative stress related to iron in a MPTP-induced PD mouse model (Xu et al., 2016). EGCG treatment has also shown to modulate autophagy and extend cell survival (Holczer et al., 2018), a mechanism that could also be involved in its neuroprotective properties of EGCG (Limanaqi et al., 2019). The effects of the treatment with a standardized **green tea extract** have also been analyzed in a preclinical model of PD (Pinto et al., 2015). In this study, long-term treatment with **green tea extract** resulted in positive effects on motor and behavioral performance of treated animals with respect to control, and in a reversal of DA levels in the striatum and protection against oxidative stress (Pinto et al., 2015). More details on the neuroprotective mechanisms of **EGCG** can be found elsewhere (Wang et al., 2022). Considering the accumulated pre-clinical evidence, the disease-modifying properties of **EGCG** should be further analyzed in clinical trials.

#### 3.1.3. Cannabinoids

**Cannabinoids** are a class of secondary metabolites found in *Cannabis sativa*, a plant whose leaves and flowers have been used since ancient times for different purposes. Cannabidiol (CBD) is the second most abundant component of the plant, behind delta9-tetrahydrocannabinol (Δ^9^-THC). The endocannabinoid system, mainly composed of CB1 and CB2 receptors, and their endogenous ligands, plays a key role in the regulation of physiological processes and its alteration has been observed in several pathological conditions, such as movement disorders (Bilkei-Gorzo, 2012; Buhmann et al., 2017, 2019). For this reason, many efforts to develop cannabinoid-based therapeutics to target the endocannabinoid system for the treatment of disease have been made, but these efforts have been challenged by the complexity and promiscuity of the cannabinoids actions (Morales and Jagerovic, 2020). In the context of PD, the role of the endocannabinoid system is still unclear (Cristino et al., 2020; Junior et al., 2020; Morales and Jagerovic, 2020). Nevertheless, treatment with different phytocannabinoids has shown neuroprotective effects in preclinical animal models of PD (García et al., 2011; Ojha et al., 2016; Espadas et al., 2020). Moreover, **Nabilone**, a synthetic analog of tetrahydrocannabinol, was included in 2 clinical trials (NCT03769896; NCT03773796, Table 2) to assess its efficacy and safety for the treatment of non-motor symptoms in patients with PD (Peball et al., 2019). Results from these studies indicate that **Nabilone** treatment has beneficial effects on sleep outcomes in PD patients that have been experiencing sleep problems at baseline (Peball et al., 2022). Whether or not phytocannabinoids or their synthetic derivatives can have a significant impact on motor performance of PD patients remains to be elucidated (Buhmann et al., 2017).

### 3.2. Polyphenolic compounds in HD

#### 3.2.1. Resveratrol

Oral treatment with **resveratrol** extended lifespan in both models and reversed motor impairment related to mHTT expression in treated mice compared to control, effects that can be explained by the activation of the ERK signaling pathway (Maher et al., 2011). ERK signaling alterations have been related to the development of movement disorders (Hutton et al., 2017) and, in turn, mHTT has shown to alter ERK signaling in HD models (Apostol et al., 2006), which might explain the neuroprotective effects of resveratrol treatment. **Resveratrol** has also shown to protect against dopamine and mHTT toxicity in a neuronal cell model of HD by preventing oxidative stress and promoting autophagy-degradation of mHTT (Vidoni et al., 2018). Also, resveratrol treatment has shown to improve mitochondrial activity in a transgenic mouse model of HD, an effect that correlated with a significant improvement in motor function of treated animals compared to control (Naia et al., 2017). The therapeutic potential of **resveratrol** was analyzed in a clinical trial comprising 102 participants with HD (NCT023366339, Table 2), where the primary output was to assess the rate of caudate atrophy after 1 year of treatment. This clinical trial was recently completed, but no results have been posted yet.

#### 3.2.2. EGCG

**EGCG** treatment inhibits early events of mHTT aggregation *in vitro*, reduces the toxicity of mHTT in a yeast model of HD, and improves motor performance in a transgenic fly model of HD (Ehrnhoefer et al., 2006). Also, treatment with a green tea infusion has shown to modestly reduce neurodegeneration related to mHTT aggregation in a fly model of HD (Varga et al., 2020). A phase 2 multicenter clinical trial of EGCG, denominated the ETON-Study (NCT01357681, Table 2), aimed to analyze the effects on cognitive function of EGCG treatment for 1 year in patients with HD and the safety and tolerability of the treatment were also studied. This study was completed in 2015, but results were not posted.

#### 3.2.3. Curcumin

**Curcumin** is one of the most active polyphenolic compounds from Curcuma, has shown to possess anti-oxidant and anti-inflammatory potential in the treatment of several disorders including diabetes, cardiovascular, neurologic, metabolic, inflammatory, and skin disorders, hepatotoxicity, respiratory tract infections, and diseases of infectious origin (Zhou et al., 2011; Khan et al., 2019). **Curcumin** treatment has been analyzed in pre-clinical models of Alzheimer's disease (AD) and amyotrophic lateral sclerosis (ALS), which have shown promising results (Chico et al., 2018; Voulgaropoulou et al., 2019; Mohseni et al., 2021; Lv et al., 2022). In these studies, curcumin treatment has shown to inhibit protein aggregation and decrease neuroinflammation, mechanisms that could also be promissory for PD and HD treatment. Moreover, curcumin treatment has also been analyzed in clinical trials of AD and ALS (Ringman et al., 2012; Chico et al., 2018; Voulgaropoulou et al., 2019; Mohseni et al., 2021; Lv et al., 2022). Furthermore, a chronic administration of curcumin in preclinical models of Huntington's disease has been shown to alleviate both the brain pathophysiology associated with reduced levels of huntingtin protein aggregates and to alleviate the motor symptoms described in R6/2 mice, along with reduced inflammation and intestinal damage associated with the progression of Huntington's disease (Elifani et al., 2019). Thus, curcumin treatment is a potential candidate for clinical trials in PD and HD patients.

#### 3.2.4. Cannabinoids

The evidence for the use of cannabinoids as treatment for HD has been somewhat mixed given the high heterogeneity of different motor and cognitive symptoms of HD patients. **Dronabinol** is a synthetic form of delta-9-tetrahydrocannabinol (Δ^9^-THC), the primary psychoactive component of cannabis. This natural product is a partial agonist at Cannabinoid-1 receptor (CB1R) and Cannabinoid-2 receptor (CB2R). In a clinical trial (NCT01502046), treatment with **dronabinol** showed beneficial symptomatic motor effects in HD patients with dystonia as a primary motor syndrome (rigidity or muscle contracture). It was also observed an improvement in weight and food intake in patients with more advanced disease stages, but **dronabinol** treatment has failed to demonstrate positive effects in the management of chorea (Saft et al., 2018).

## 4. Repurposing FDA-approved drugs for PD and HD treatment

Considering that the translation from the accumulated knowledge on human disease to successful therapeutics has been slower than expected, repurposing drugs has become an attractive strategy for the identification of new therapeutic uses of already approved drugs (Oprea and Mestres, 2012; Chen et al., 2019; Pushpakom et al., 2019; Roessler et al., 2021; Koponen et al., 2022). This has become especially important for those diseases that, like neurodegenerative diseases, have an unmet clinical need. The advantages of drug repurposing are diverse: the failure risk is lower, the timeframe for development is reduced, and less investment is needed, as the candidate drugs have already been developed and their safety and tolerability has been assessed in clinical trials (Pushpakom et al., 2019). Drug repurposing strategies and examples of successful drug repurposing can be revised on (Pushpakom et al., 2019). In this section we will discuss examples of drugs that have been repurposed or whose repurposing is being studied for the treatment of PD and HD (Figure 1C).

### 4.1. Drug repurposing in PD

As mentioned before, the gold standard pharmacological intervention for PD is **L-dopa**, a dopamine agonist, which can be combined with MAO-B and COMT inhibitors to improve its efficacy and lower its side effects. Since its discovery and initial use for PD treatment, just a few drugs have accomplished to be approved by the FDA, and most of them share its mechanism of action. The imperative need to expand the catalog of drugs available for patients has opened the opportunity for the study of previous FDA-approved drugs. This was the case back in the 1970s for **amantadine**, a NMDA receptor antagonist drug that was originally developed as an influenza prophylactic and later, after appropriate clinical trials, repurposed as treatment for motor complications in PD treatment (Athauda and Foltynie, 2018). Thereafter, several drugs have been tested for its positive effects in PD patients. **Ambroxol** is an expectorant drug that has been available worldwide for more than 30 years. After a high-throughput *screen* of an FDA-approved drugs library comprising 1,040 different compounds, **ambroxol** was identified and found to be a pH-dependent, mixed-type inhibitor of glucocerebrosidase (GCase), interacting with both active and non-active site residues of GCase. Its inhibitory activity was maximal at neutral pH in endoplasmic reticulum (ER), and the activity was undetectable at the acidic pH of lysosome (Maegawa et al., 2009). Ambroxol acts as a pharmacological chaperone with the ability to stabilize the GCase (Maegawa et al., 2009). GCase has been identified as a potential pharmacological target for the treatment of PD as mutations in its gene is one of the strongest genetic factors for PD (Avenali et al., 2020), and thus, **ambroxol** properties have been analyzed in different models of PD (Athauda and Foltynie, 2018). *In vivo* data indicated that ambroxol could cross the blood-brain barrier and reduce the levels of α-syn and p-α-syn in brain mice (Migdalska-Richards et al., 2016). Also, **ambroxol** treatment has recently demonstrated its safety and tolerability in PD patients with and without GBA1 gene mutations (Mullin et al., 2020), and is currently under clinical trial to demonstrate its disease-modifying properties in PD patients by the evaluation of cognitive and motor test, also the detection of neurodegeneration markers by MRI, the determination of pharmacokinetics and pharmacodynamics of ambroxol in plasma and the evaluation of GCase activity in lymphocytes (Silveira et al., 2019) (NCT052875, Table 3).

**Table 3:** Description of repurposed FDA-approved drugs for Parkinson's disease of Huntington's disease.

			**Clinical trials**							
**Name**	**Target**	**Preclinical models (references)**	**CT identifier**	**Phase**	**First posted**	**Primary purpose**	**Population**	**Study design**	**Primary outcomes**	**Results**
**Ambroxol**	Chaperone that stabilize the glucocerebrosidase (GCase) protein	PD mice and nonhuman primate models (Hutton et al., 2017)	NCT05287503	Phase 2	2022	Treatment	PD patients	60 participants	Change from baseline in Montreal Cognitive Assessment score. This 30-point test investigates global cognitive functions and it has been recommended for the assessment of Parkinson's dementia. The lower the score the worse the cognitive functions [Time frame: baseline and week 52]. Change from baseline in conversion rate from normal cognitive function (PD-N) to mild cognitive impairment (PD-MCI) and from PD-N or PD-MCI to Parkinson-Dementia (PD-D). Rate of conversion from normal cognitive status to MCI or from MCI to overt dementia over the 52-week treatment period [Time frame: baseline and week 52].	No study results posted
**Isradipine**	Calcium channel blocker	PD mice models (Hutton et al., 2017)	NCT00909545; NCT00753636; NCT02168842	Phase 2; Phase 2; Phase 3	2009; 2008; 2014	Treatment	PD patients	99 participants; 31 participants; 336 participants	Tolerability of the three dosages (5, 10, and 20 mg) of Isradipine CR. Tolerability will be judged by the proportion of subjects enrolled in a dosage group able to complete the 12 month study or to the time of initiation of dopaminergic therapy on their original assigned dosage. Tolerability of each active arm will be compared to placebo group [Time frame: Baseline to 12 months or the time to require dopaminergic therapy]; Tolerability of isradipine based on the number of participants that complete the study [Time frame: 1 year]; adjusted mean change in total unified Parkinson's disease rating scale (UPDRS) score. Efficacy of isradipine to slow progression of Parkinson's disease disability to be determined by the change in the total (Part I-III) UPDRS score in the active treatment arm vs. placebo between the baseline and 36 month visit. The change of UPDRS ranges from −30 to 80, larger value shows more disability from PD [Time frame: baseline to 36 months of treatment]. Adjusted mean change in adjusted UPDRS Score. Efficacy of isradipine to slow progression of Parkinson's disease disability to be determined by the change in the adjusted UPDRS Score in the active treatment arm vs. placebo between the baseline and 36 month visit. The change of adjusted UPDRS ranges from−100 to 150, larger value shows more disability from PD [Time frame: baseline to 36 months of treatment]	Safety, tolerability and efficacy as already been tested, but its properties as a disease modifying treatment for PD remain to be analyzed
**Deferiprone**	Iron chelator	PD rats models (Voulgaropoulou et al., 2019; Koponen et al., 2022)	NCT02655315	Phase 2	2016	Treatment	PD patients	372 participants	Global effect (symptomatic and disease modifying effects) on motor and non motor handicap. The change in the total movement disorders society-unified Parkinson disease rating scale score between baseline and 36 weeks (i.e. the end of the placebo-controlled phase for analysis of both disease-modifying and symptomatic effects) [Time frame: at 36 weeks]	No study results posted
**Clenbuterol**	B2AR agonist	PD mice models (Chen et al., 2019)								
**Salbutamol**	B2AR agonist	PD rats models (Athauda and Foltynie, 2018)								
**Fenofibrate**	Activation of peroxisome proliferator-activated receptor-β (PPAR-β)	HD mice models (Becker et al., 2008)	NCT03515213	Phase 2	2018	Treatment	HD patients	20 participants	Change in PGC-1alpha from baseline to month 6. Change in PGC-1alpha from baseline to month 6 [Time frame: baseline, 1, 2, 3, 4, 5, and 6 months]	No study results posted
**KD3010**	PPAR-δ agonist	HD mice models (Silveira et al., 2019)								
**Metformin**	Activation of AMPK	HD mice models (Dexter et al., 2011; Martin-Bastida et al., 2017; Hider and Hoffbrand, 2018)	NCT04826692	Phase 3	2021	Treatment	HD patients	60 participants	Evaluate the effect of metformin on the scores obtained in different cognitive subtests that make up the unified Huntington's disease rating scale [Time frame: baseline—week 52]	No study results posted

An epidemiological study showed that long term treatment with calcium channel blocker antihypertensives was related with a lower risk of developing PD (Becker et al., 2008) and, due to its affinity for calcium channels and brain bioavailability, **isradipine** was considered as a promissory candidate for PD treatment (Athauda and Foltynie, 2018). The safety and tolerability of **isradipine** treatment have already been analyzed in clinical trials (NCT00909545, NCT00753636, Table 3) but recently, the STEADY-PD III trial which studied the efficacy of **isradipine** treatment in early PD, concluded that this intervention did not slow the progression of the disease (NCT02168842, Table 3) (Parkinson Study Group STEADY-PD III Investigators., 2020).

Another drug that is being studied to be repurposed for PD treatment is **deferiprone**, an iron chelator that can cross the blood brain barrier (BBB) and remove the excess of iron in the brain (Dexter et al., 2011; Martin-Bastida et al., 2017). This drug was initially designed in the 1980s for the treatment of iron overload related to blood transfusions (Hider and Hoffbrand, 2018). More recently, it has been observed that iron can accumulate in the brain during aging, mediating ROS production and facilitating protein aggregation and neuronal death (Guo and Scarlata, 2013; Joppe et al., 2019; Lévy et al., 2019). Hence **deferiprone** might have effects over α-syn aggregation. Furthermore, as iron is required for dopamine synthesis, its concentrations are higher in substantia nigra pars compacta (SNpc) dopaminergic neurons and, as these neurons are more susceptible to the higher production of ROS associated to aging (Choi et al., 2012; Trist et al., 2019), the beneficial effects of **deferiprone** in the treatment of PD might also be directly related to its iron chelation activity (Devos et al., 2020). Thus, **deferiprone** treatment has been analyzed in order to assess its neuroprotective properties in preclinical models of PD, observing that the subcutaneous administration of **deferiprone** can rescue dopaminergic neuron from degeneration (Dexter et al., 2011) and that its oral administration can improve motor and cognitive performance in treated animals, without having a significative effect over α-syn aggregation (Carboni et al., 2017). Considering the preclinical data, **deferiprone** treatment has also been analyzed in clinical trials including PD patients. In a phase 2 clinical trial (NCT02655315, Table 3), the short-term treatment with **deferiprone** in patients with early-stage PD showed to be safe, well tolerated, and able to reduce iron accumulation in specific brain regions (Martin-Bastida et al., 2017). The evidence accumulated through the years led to the development of a large multicenter clinical trial which is currently evaluating the disease-modifying properties of **deferiprone** on 372 subjects (NCT02655315, Table 3). To date, no results regarding this trial have been published.

An important line of investigation respecting drug repurposing for PD is related to inhibiting or decreasing the α-syn aggregation. In 2017, Mittal et al., uncovered a novel function of a group of drugs previously approved by the FDA. Under the premise of finding drugs that can reduce the levels of the SNCA gene, and hence, the α-syn levels, a **selective** **β2-adrenoreceptor (β2AR)** agonists reduced the expression levels of SNCA (Mittal et al., 2017). The decrease in the expression of SNCA and the α-syn levels using β2AR agonists was confirmed using cell lines, primary neurons, and animal models (Mittal et al., 2017). Interestingly, using mice treated with MPTP, a chemical used to model PD, the investigators found that treatment of these mice with **clenbuterol**, a β2AR agonist, abrogated the neurodegeneration observed under MPTP administration (Mittal et al., 2017). Moreover, these findings were confirmed using iPSCs-derived neuronal cultures from a PD patient (Mittal et al., 2017). Interestingly, the use of **salbutamol**, a β2AR agonist and a drug typically prescribed for asthma, was associated with a reduced risk of PD in the Norwegian population (Mittal et al., 2017). The later finding was also confirmed in a study using medical records of a large Israeli cohort (Gronich et al., 2018). **Salbutamol** has also been linked in the promotion of the expression of fibroblast growth factor 20 (FGF20) in the striatum of naïve rats (Fletcher et al., 2019). FGF20 is a brain-specific factor that plays a crucial role in the development, maintenance, and survival of dopaminergic neurons. Interestingly, **salbutamol** increased dopaminergic neuron survival in 6-OHDA-lesioned rats (Fletcher et al., 2019), supporting the results of Mittal et al. (2017). Despite these interesting results, other have found no association between the use of β2AR agonists and PD risk (Searles Nielsen et al., 2018), so further studies are needed to rather confirm or disprove these findings. Moreover, to date no clinical trial for the study of the association of salbutamol use and PD risk is under way.

### 4.2. Drug repurposing in HD

Several approved drugs have been analyzed for the treatment of HD and some of them have been included in clinical trials, but just a few currently remain under active development (Devadiga and Bharate, 2022). **Fenofibrate** is an FDA-approved drug that is commonly used for the treatment of severe hypertriglyceridemia and mixed dyslipidemia. **Fenofibrate** can promote the activation of peroxisome proliferator-activated receptor-α (PPAR-α), a subtype of PPAR receptors expressed in brown adipose tissue, liver, kidney, heart, skeletal muscle, CNS, and T-cells, that acts as a lipid sensor performing essential metabolic functions (McKeage and Keating, 2011; Bhateja et al., 2012; Dickey et al., 2016). PPAR-α activation has been related to anti-inflammatory and antioxidant effects, and thus, the neuroprotective effects of PPAR-α activators such as fenofibrate have been investigated (Bordet et al., 2006; Bhateja et al., 2012). In this context, it has been observed that the intraperitoneal administration of **fenofibrate** significantly improved motor deficits and cognitive function, reduced oxidative stress, and restored antioxidant mechanisms in a pharmacologically-induced model of HD (Bordet et al., 2006). A phase II clinical trial evaluating the safety and efficacy of **fenofibrate** treatment was initiated in 2017 and remains active (NCT03515213, Table 3). Results were expected to be posted on august 2021 but, to date, they have not been reported. It has also been reported that HTT can directly interact with PPAR-δ, the most abundant PPAR in the CNS, altering its function as a transcriptional factor and, thus, contributing to mitochondrial dysfunction and neurotoxicity in pre-clinical models of HD (Dickey et al., 2016). Furthermore, altering the activation of PPAR-δ through the expression of a transgenic construct in mice induces HD-like phenotypes, confirming the crucial role of this receptor in maintaining neuronal health (Dickey et al., 2016). Consequently, intraperitoneal administration of the selective and potent PPAR-δ agonist **KD3010** attenuated neurological dysfunction and improved motor function in HD mice without any notable side effects (Dickey et al., 2016). Thus, PPAR activators hold potential for the development of new therapeutics for NMDs and other neurological diseases either through repurposing or discovery of new molecules.

**Metformin** is another FDA approved drug that is used worldwide for the treatment of type 2 diabetes mellitus. In 2017, results from an observational study on HD revealed that metformin intake is associated with a better cognitive function in HD patients (Hervás et al., 2017). These observations were in line with previous reports that indicated that metformin treatment had protective effects in diverse pre-clinical models of HD (Ma et al., 2007; Jin et al., 2016; Vázquez-Manrique et al., 2016). The underlying mechanisms of the neuroprotective effects of metformin in HD might be related to its effects on the AMP-activated protein kinase (AMPK) but also other mechanisms might be involved (Ma et al., 2007; Jin et al., 2016; Vázquez-Manrique et al., 2016; Sanchis et al., 2019; Río et al., 2022). AMPK plays a key role in the response to metabolic changes, targeting proteins in diverse catabolic pathways to inactivate them and activating anabolic pathways to restore homeostasis (Vázquez-Manrique et al., 2016). Using a transgenic mouse model of HD, Ma et al. (2007) showed that the oral administration of metformin was associated to a significant increment in AMPK activation in the brain of treated animals and this, in turn, was related to increased survival and decreased hind limb clasping time in male mice, which is representative of an improvement in disease progression compared to control. Furthermore, it has been observed that AMPK activation by metformin treatment protects against neuronal dysfunction induced by overexpressing mHTT in *Caenorhabditis elegans* and mice (Ma et al., 2007). Moreover, Sanchis et al. (2019) demonstrated that metformin treatment reduced neuronal toxicity and improved neuropsychiatric and motor behavior in a transgenic mouse model of HD that overexpresses mHTT. Importantly, they also observed that metformin could cross the blood-brain barrier, reducing mHTT aggregation, preventing inflammation, and decreasing the levels of PERK, a biomarker of cellular stress (Sanchis et al., 2019). Other mechanisms involved in the neuroprotective effects of metformin can be revised elsewhere (Río et al., 2022). Considering this evidence, a clinical trial to analyze the safety and efficacy of metformin in adults with HD was initiated by the end of 2021 (NCT04826692). Results from this interventional study are expected to be posted by august 2024.

## 5. Discussion

The most studied motor disorders are PD and HD. These NMDs are characterized by different etiological factors, differential vulnerability, the development of motor symptoms, and specific clinical manifestations. Interestingly, the neuronal circuits affected in these pathologies are common and thus, this knowledge could drive the recognition of the potential molecular pathways to develop future therapeutic treatments. We explored this hypothesis in this review, and, through extensive literature research, we noticed that common molecular pathways came up as interesting targets to evaluate and develop new small molecules that modulate specific molecular processes, such as protein aggregation, inflammation, and G protein-coupled receptor signaling. Nevertheless, current clinical trials in NMDs have a high failure rate. This might be due to patients presenting a high diversity in both genetic backgrounds and exposure of patients to environmental factors, which might influence the beginning of symptoms manifestation. For this reason, it is necessary to search for new biomarkers that could help us to discriminate between different subpopulations of patients, to reach a more personalized and precise medicine. This could allow for a better selection of participants included in clinical trials, considering the effect of the small molecules being studied on specific molecular processes.

In the context of natural products, a limitation in the development of these molecules as therapeutic agents for PD, HD, and other diseases, appears to be their lack of specificity. As stated before, compounds like resveratrol, EGCG and curcumin have been extensively investigated and they are normally recognized as hits in multiple assays. This has led to the recognition of molecular targets for these compounds, some of which have been discussed here. Compounds with this promiscuous nature have been denominated Pan Assay Interference compounds (PAINS) and are recognized as undesirable compounds in drug discovery efforts (Baell and Holloway, 2010; Baell, 2016). Some structural features on different natural products have been identified as PAINS motifs, like the catechol groups in EGCG and the quinone in the oxidized form of resveratrol (Baell, 2016), which might explain the diversity of mechanisms related to their pharmacological effects. Furthermore, although natural products are considered safe for consuming, these molecules can produce adverse effects by, for example, pharmacological interactions with other drugs (Wang et al., 2020). Despite these limitations, natural products are still a valuable starting point for drug development, as they can be used as scaffolds for the rational design of new drugs with the aid of high-content screening and computational tools (Kurita et al., 2015; Rodrigues et al., 2016; Davison and Brimble, 2019).

An important limitation for small molecules, either synthetic or from natural origin, in the context of NMD treatment, is the ability of these compounds to reach the central nervous system. Considering the molecular pathways involved in the development of NMDs, the bioavailability of the drug in the brain is a key factor to consider when developing an effective therapy for these pathologies. This limitation might be already accounted for when screening large libraries of synthetic molecules, as the physicochemical characteristics of the compounds can be previously optimized to assure their distribution to the brain (Wan et al., 2007). The application of recent advances in molecular docking and computer-aided design combined with cell-based platforms are helping to advance in the discovery of already clinically applicable molecules (Aldewachi et al., 2021). In the context of natural products, brain accessibility is also an issue. As an example, the oral bioavailability, of polyphenolic compounds has been pointed out by researchers as a limitation for the use of these molecules as pharmacological products (D'Archivio et al., 2010; Bohn, 2014; Kawabata et al., 2019). Nevertheless, evidence for the accumulation of these molecules in the brain can be found elsewhere (Ehrnhoefer et al., 2006; Figueira et al., 2017; Maher, 2019). Furthermore, advances in pharmaceutical technology, such as biomaterial and nanotechnology applications, have also been applied by researchers to overcome the physiological barriers that limit accessibility of drugs to the brain (Saeedi et al., 2019, 2022; Teleanu et al., 2019).

It is important to also recognize the importance of biotechnological products in drug discovery. Biotechnological products have also emerged as a promising class of drugs for the treatment of neurodegenerative diseases. An example of this is the engineered fusion protein **NPT088**. **NPT088** targets amyloid *in vitro* and in animal models of Alzheimer's disease (AD), reducing β-amyloid plaque and tau aggregate loads in a mouse disease model (Levenson et al., 2016). In a clinical trial, patients with mild to moderate AD, were treated with **NTP088**. At the endpoint (6 months), **NTP088** treatment was generally safe and well-tolerated (Michelson et al., 2019) but no significant effects over brain plaques, tau aggregates or AD symptoms were observed. However, it is important to notice that NPT088 more specifically targets aggregates of misfolded proteins rather than monomer subunits and consequently, it might be interesting to evaluate this type of drugs in the context of other misfolded protein-related neurodegenerative disorders.

Thus, there is hope for the discovery of new or old molecules that can be used in the treatment of NMDs and for the development of drugs that can be easily and effectively administered to patients.

## 6. Concluding remarks

Although we still have no cure for most NMDs, current research involving new molecules with novel mechanisms of action is promising, but not free of limitations. Considering the broad spectrum of research aiming to decipher the molecular mechanisms underlying NMDs, the number of new or repurposed molecules that reach clinical trials is still low. This might be explained, to some extent, by the intrinsic limitations of animal models for preclinical studies, the difficulty of accessing the brain, and the lack of human biomarkers that strongly correlate to the progress of the disease, and the difficulty of accessing the brain. Nonetheless, recent advances in strategies to accelerate the discovery or repurposing of drugs, on top of the innovations on delivery systems to the brain, restore the confidence that small molecules can play big roles in the treatment of NMDs.

## Author contributions

RP-A, DS, PT-E, MC-O, and RV wrote and edited the manuscript and planned the manuscript. RV prepared the figures. All authors contributed equally to the critical reading of the final manuscript, including figures.

## References

[B1] Achenbach J. Saft C. (2021). Another perspective on Huntington's disease: disease burden in family members and pre-manifest HD when compared to genotype-negative participants from ENROLL-HD. Brain Sci. 11, 1621. 10.3390/brainsci11121621 34942923 PMC8699274

[B2] Aldewachi H. Al-Zidan R. N. Conner M. T. Salman M. M. (2021). High-throughput screening platforms in the discovery of novel drugs for neurodegenerative diseases. Bioengineering 8, 30. 10.3390/bioengineering8020030 33672148 PMC7926814

[B3] Ammal Kaidery N. Tarannum S. Thomas B. (2013). Epigenetic landscape of Parkinson's disease: emerging role in disease mechanisms and therapeutic modalities. Neurotherapeutics 10, 698–708. 10.1007/s13311-013-0211-8 24030213 PMC3805874

[B4] Apostol B. L. Illes K. Pallos J. Bodai L. Wu J. Strand A.. (2006). Mutant huntingtin alters MAPK signaling pathways in PC12 and striatal cells: ERK1/2 protects against mutant huntingtin-associated toxicity. Hum. Mol. Genet. 15, 273–285. 10.1093/hmg/ddi443 16330479

[B5] Arbo B. D. André-Miral C. Nasre-Nasser R. G. Schimith L. E. Santos M. G. Costa-Silva D.. (2020). Resveratrol derivatives as potential treatments for Alzheimer's and Parkinson's disease. Front. Aging Neurosci. 12, 103. 10.3389/fnagi.2020.00103 32362821 PMC7180342

[B6] Athauda D. Foltynie T. (2018). Drug repurposing in Parkinson's disease. CNS Drugs. 32, 747–761. 10.1007/s40263-018-0548-y 30066310

[B7] Avenali M. Blandini F. Cerri S. (2020). Glucocerebrosidase defects as a major risk factor for Parkinson's disease. Front. Aging Neurosci. 12, 97. 10.3389/fnagi.2020.00097 32372943 PMC7186450

[B8] Azam S. Haque M. E. Balakrishnan R. Kim I.-S. Choi D.-K. (2021). The ageing brain: molecular and cellular basis of neurodegeneration. Front. Cell Dev. Biol. 9, 683459. 10.3389/fcell.2021.683459 34485280 PMC8414981

[B9] Baell J. B. (2016). Feeling nature's PAINS: natural products, natural product drugs, and pan assay interference compounds (PAINS). J. Nat. Prod. 79, 616–628. 10.1021/acs.jnatprod.5b00947 26900761

[B10] Baell J. B. Holloway G. A. (2010). New substructure filters for removal of pan assay interference compounds (PAINS) from screening libraries and for their exclusion in bioassays. J. Med. Chem. 53, 2719–2740. 10.1021/jm901137j 20131845

[B11] Bakoyiannis I. Daskalopoulou A. Pergialiotis V. Perrea D. (2019). Phytochemicals and cognitive health: are flavonoids doing the trick? Biomed. Pharmacother. 109, 1488–1497. 10.1016/j.biopha.2018.10.086 30551400

[B12] Barth A. L. Schneider J. S. Johnston T. H. Hill M. P. Brotchie J. M. Moskal J. R.. (2020). NYX-458 improves cognitive performance in a primate Parkinson's disease model. Mov. Disord. 35, 640–649. 10.1002/mds.27962 31967361

[B13] Bastianetto S. Menard C. Quirion R. (2015). Neuroprotective action of resveratrol. Biochim. Biophys. Acta 1852, 1195–1201. 10.1016/j.bbadis.2014.09.011 25281824

[B14] Becker C. Jick S. S. Meier C. R. (2008). Use of antihypertensives and the risk of Parkinson disease. Neurology 70, 1438–1444. 10.1212/01.wnl.0000303818.38960.44 18256367

[B15] Bhateja D. K. Dhull D. K. Gill A. Sidhu A. Sharma S. Reddy B. V. K.. (2012). Peroxisome proliferator-activated receptor-alpha activation attenuates 3-nitropropionic acid induced behavioral and biochemical alterations in rats: possible neuroprotective mechanisms. Eur. J. Pharmacol. 674, 33–43. 10.1016/j.ejphar.2011.10.029 22056833

[B16] Bilkei-Gorzo A. (2012). The endocannabinoid system in normal and pathological brain ageing. Philos. Trans. R. Soc. Lond. B. Biol. Sci. 367, 3326–3341. 10.1098/rstb.2011.0388 23108550 PMC3481530

[B17] Bohn T. (2014). Dietary factors affecting polyphenol bioavailability. Nutr. Rev. 72, 429–452. 10.1111/nure.12114 24828476

[B18] Bordet R. Gel,é P. Duriez P. Fruchart J.-C. (2006). PPARs: a new target for neuroprotection. J. Neurol. Neurosurg. Psychiatry 77, 285–287. 10.1136/jnnp.2005.077495 16484630 PMC2077704

[B19] Buhmann C. Mainka T. Ebersbach G. Gandor F. (2017). Cannabinoids in Parkinson's disease. Cannabis Cannabinoid Res. 2, 21–29. 10.1089/can.2017.0002 28861502 PMC5436333

[B20] Buhmann C. Mainka T. Ebersbach G. Gandor F. (2019). Evidence for the use of cannabinoids in Parkinson's disease. J. Neural Transm. 126, 913–924. 10.1007/s00702-019-02018-8 31131434

[B21] Cacabelos R. (2017). Parkinson's disease: from pathogenesis to pharmacogenomics. Int. J. Mol. Sci. 18, 551. 10.3390/ijms18030551 28273839 PMC5372567

[B22] Carboni E. Tatenhorst L. Tönges L. Barski E. Dambeck V. Bähr M.. (2017). Deferiprone rescues behavioral deficits induced by mild iron exposure in a mouse model of alpha-synuclein aggregation. Neuromolecular Med. 19, 309–321. 10.1007/s12017-017-8447-9 28623611 PMC5570801

[B23] Carrera I. Cacabelos R. (2019). Current drugs and potential future neuroprotective compounds for Parkinson's disease. Curr. Neuropharmacol. 17, 295–306. 10.2174/1570159X17666181127125704 30479218 PMC6425078

[B24] Chen K.-P. Hua K.-F. Tsai F.-T. Lin T.-Y. Cheng C.-Y. Yang D.-I.. (2022). A selective inhibitor of the NLRP3 inflammasome as a potential therapeutic approach for neuroprotection in a transgenic mouse model of Huntington's disease. J. Neuroinflammation 19, 56. 10.1186/s12974-022-02419-9 35219323 PMC8882273

[B25] Chen X. Gumina G. Virga K. G. (2019). Recent advances in drug repurposing for Parkinson's disease. Curr. Med. Chem. 26, 5340–5362. 10.2174/0929867325666180719144850 30027839

[B26] Cheng L. Quek C. Y. J. Hung L. W. Sharples R. A. Sherratt N. A. Barnham K. J.. (2016). Gene dysregulation is restored in the Parkinson's disease MPTP neurotoxic mice model upon treatment of the therapeutic drug Cu(II)(atsm). Sci. Rep. 6, 22398. 10.1038/srep22398 26928495 PMC4772163

[B27] Cherny R. A. Ayton S. Finkelstein D. I. Bush A. I. McColl G. Massa S. M.. (2012). PBT2 Reduces toxicity in a *C. elegans* model of polyQ aggregation and extends lifespan reduces striatal atrophy and improves motor performance in the R6/2 mouse model of Huntington's disease. J. Huntingtons. Dis. 1, 211–219. 10.3233/JHD-120029 25063332

[B28] Chico L. Ienco E. C. Bisordi C. Gerfo A. L. Petrozzi L. Petrucci A.. (2018). Amyotrophic lateral sclerosis and oxidative stress: a double-blind therapeutic trial after curcumin supplementation. CNS Neurol. Disord. Drug Targets 17, 767–779. 10.2174/1871527317666180720162029 30033879

[B29] Choi D.-H. Cristóvão A. C. Guhathakurta S. Lee J. Joh T. H. Beal M. F.. (2012). NADPH oxidase 1-mediated oxidative stress leads to dopamine neuron death in Parkinson's disease. Antioxid. Redox Signal. 16, 1033–1045. 10.1089/ars.2011.3960 22098189 PMC3315177

[B30] Choudhary S. Kumar P. Malik J. (2013). Plants and phytochemicals for Huntington's disease. Pharmacogn. Rev. 7, 81–91. 10.4103/0973-7847.120505 24347915 PMC3841999

[B31] Commenges D. Scotet V. Renaud S. Jacqmin-Gadda H. Barberger-Gateau P. Dartigues J. F.. (2000). Intake of flavonoids and risk of dementia. Eur. J. Epidemiol. 16, 357–363. 10.1023/A:1007614613771 10959944

[B32] Crichton G. E. Bryan J. Murphy K. J. (2013). Dietary antioxidants, cognitive function and dementia–a systematic review. Plant Foods Hum. Nutr. 68, 279–292. 10.1007/s11130-013-0370-0 23881465

[B33] Cristino L. Bisogno T. Di Marzo V. (2020). Cannabinoids and the expanded endocannabinoid system in neurological disorders. Nat. Rev. Neurol. 16, 9–29. 10.1038/s41582-019-0284-z 31831863

[B34] D'Archivio M. Filesi C. Varì R. Scazzocchio B. Masella R. (2010). Bioavailability of the polyphenols: status and controversies. Int. J. Mol. Sci. 11, 1321–1342. 10.3390/ijms11041321 20480022 PMC2871118

[B35] Dash R. Jahan I. Ali M. C. Mitra S. Munni Y. A. Timalsina B.. (2021). Potential roles of natural products in the targeting of proteinopathic neurodegenerative diseases. Neurochem. Int. 145, 105011. 10.1016/j.neuint.2021.105011 33711400

[B36] Davison E. K. Brimble M. A. (2019). Natural product derived privileged scaffolds in drug discovery. Curr. Opin. Chem. Biol. 52, 1–8. 10.1016/j.cbpa.2018.12.007 30682725

[B37] Decressac M. Volakakis N. Björklund A. Perlmann T. (2013). NURR1 in Parkinson disease–from pathogenesis to therapeutic potential. Nat. Rev. Neurol. 9, 629–636. 10.1038/nrneurol.2013.209 24126627

[B38] Devadiga S. J. Bharate S. S. (2022). Recent developments in the management of Huntington's disease. Bioorg. Chem. 120, 105642. 10.1016/j.bioorg.2022.105642 35121553

[B39] Devore E. E. Grodstein F. van Rooij F. J. A. Hofman A. Stampfer M. J. Witteman J. C. M.. (2010). Dietary antioxidants and long-term risk of dementia. Arch. Neurol. 67, 819–825. 10.1001/archneurol.2010.144 20625087 PMC2923546

[B40] Devos D. Cabantchik Z. I. Moreau C. Danel V. Mahoney-Sanchez L. Bouchaoui H.. (2020). Conservative iron chelation for neurodegenerative diseases such as Parkinson's disease and amyotrophic lateral sclerosis. J. Neural Transm. 127, 189–203. 10.1007/s00702-019-02138-1 31912279

[B41] Dexter D. T. Statton S. A. Whitmore C. Freinbichler W. Weinberger P. Tipton K. F.. (2011). Clinically available iron chelators induce neuroprotection in the 6-OHDA model of Parkinson's disease after peripheral administration. J. Neural Transm. 118, 223–231. 10.1007/s00702-010-0531-3 21165659

[B42] Di Meo F. Valentino A. Petillo O. Peluso G. Filosa S. Crispi S.. (2020). Bioactive polyphenols and neuromodulation: molecular mechanisms in neurodegeneration. Int. J. Mol. Sci. 21, 2564. 10.3390/ijms21072564 32272735 PMC7178158

[B43] Diaz-Gerevini G. T. Repossi G. Dain A. Tarres M. C. Das U. N. Eynard A. R.. (2016). Beneficial action of resveratrol: how and why? Nutrition 32, 174–178. 10.1016/j.nut.2015.08.017 26706021

[B44] Dickey A. S. Pineda V. V. Tsunemi T. Liu P. P. Miranda H. C. Gilmore-Hall S. K.. (2016). PPAR-delta is repressed in Huntington's disease, is required for normal neuronal function and can be targeted therapeutically. Nat. Med. 22, 37–45. 10.1038/nm.4003 26642438 PMC4752002

[B45] Eddings C. R. Arbez N. Akimov S. Geva M. Hayden M. R. Ross C. A.. (2019). Pridopidine protects neurons from mutant-huntingtin toxicity *via* the sigma-1 receptor. Neurobiol. Dis. 129, 118–129. 10.1016/j.nbd.2019.05.009 31108174 PMC6996243

[B46] Ehrnhoefer D. E. Duennwald M. Markovic P. Wacker J. L. Engemann S. Roark M.. (2006). Green tea (-)-epigallocatechin-gallate modulates early events in huntingtin misfolding and reduces toxicity in Huntington's disease models. Hum. Mol. Genet. 15, 2743–2751. 10.1093/hmg/ddl210 16893904

[B47] Elifani F. Amico E. Pepe G. Capocci L. Castaldo S. Rosa P.. (2019). Curcumin dietary supplementation ameliorates disease phenotype in an animal model of Huntington's disease. Hum. Mol. Genet. 28, 4012–4021. 10.1093/hmg/ddz247 31630202

[B48] Elkouzi A. Vedam-Mai V. Eisinger R. S. Okun M. S. (2019). Emerging therapies in Parkinson disease - repurposed drugs and new approaches. Nat. Rev. Neurol. 15, 204–223. 10.1038/s41582-019-0155-7 30867588 PMC7758837

[B49] Espadas I. Keifman E. Palomo-Garo C. Burgaz S. García C. Fernández-Ruiz J.. (2020). Beneficial effects of the phytocannabinoid Delta(9)-THCV in L-DOPA-induced dyskinesia in Parkinson's disease. Neurobiol. Dis. 141, 104892. 10.1016/j.nbd.2020.104892 32387338

[B50] Figueira I. Menezes R. Macedo D. Costa I. Dos Santos C. N. (2017). Polyphenols beyond barriers: a glimpse into the brain. Curr. Neuropharmacol. 15, 562–594. 10.2174/1570159X14666161026151545 27784225 PMC5543676

[B51] Flanagan E. Müller M. Hornberger M. Vauzour D. (2018). Impact of flavonoids on cellular and molecular mechanisms underlying age-related cognitive decline and neurodegeneration. Curr. Nutr. Rep. 7, 49–57. 10.1007/s13668-018-0226-1 29892788 PMC5960493

[B52] Fletcher E. J. R. Jamieson A. D. Williams G. Doherty P. Duty S. (2019). Targeted repositioning identifies drugs that increase fibroblast growth factor 20 production and protect against 6-hydroxydopamine-induced nigral cell loss in rats. Sci. Rep. 9, 8336. 10.1038/s41598-019-44803-1 31171821 PMC6554393

[B53] Fox S. H. Katzenschlager R. Lim S.-Y. Barton B. de Bie R. M. A. Seppi K.. (2018). International Parkinson and movement disorder society evidence-based medicine review: update on treatments for the motor symptoms of Parkinson's disease. Mov. Disord. 33, 1248–1266. 10.1002/mds.27372 29570866

[B54] García C. Palomo-Garo C. García-Arencibia M. Ramos J. Pertwee R. Fernández-Ruiz J.. (2011). Symptom-relieving and neuroprotective effects of the phytocannabinoid Delta(9)-THCV in animal models of Parkinson's disease. Br. J. Pharmacol. 163, 1495–1506. 10.1111/j.1476-5381.2011.01278.x 21323909 PMC3165958

[B55] Garcia-Miralles M. Geva M. Tan J. Y. Yusof N. A. B. M. Cha Y. Kusko R.. (2017). Early pridopidine treatment improves behavioral and transcriptional deficits in YAC128 Huntington disease mice. JCI Insight 2, e95665. 10.1172/jci.insight.95665 29212949 PMC5752291

[B56] Gildawie K. R. Galli R. L. Shukitt-Hale B. Carey A. N. (2018). Protective effects of foods containing flavonoids on age-related cognitive decline. Curr. Nutr. Rep. 7, 39–48. 10.1007/s13668-018-0227-0 29892789

[B57] Gomes B. A. Q. Silva J. P. B. Romeiro C. F. R. Dos Santos S. M. Rodrigues C. A. Gonçalves P. R.. (2018). Neuroprotective mechanisms of resveratrol in Alzheimer's disease: role of SIRT1. Oxid. Med. Cell. Longev. 2018, 8152373. 10.1155/2018/8152373 30510627 PMC6232815

[B58] Gordon R. Albornoz E. A. Christie D. C. Langley M. R. Kumar V. Mantovani S.. (2018). Inflammasome inhibition prevents alpha-synuclein pathology and dopaminergic neurodegeneration in mice. *Sci. Transl*. Med. 10, eaah4066. 10.1126/scitranslmed.aah4066 30381407 PMC6483075

[B59] Grachev I. D. Meyer P. M. Becker G. A. Bronzel M. Marsteller D. Pastino G.. (2021). Sigma-1 and dopamine D2/D3 receptor occupancy of pridopidine in healthy volunteers and patients with Huntington disease: a [(18)F] fluspidine and [(18)F] fallypride PET study. Eur. J. Nucl. Med. Mol. Imaging 48, 1103–1115. 10.1007/s00259-020-05030-3 32995944 PMC8041674

[B60] Gronich N. Abernethy D. R. Auriel E. Lavi I. Rennert G. Saliba W.. (2018). beta2-adrenoceptor agonists and antagonists and risk of Parkinson's disease. Mov. Disord. 33, 1465–1471. 10.1002/mds.108 30311974

[B61] Group H. S. Frank S. Testa C. M. Stamler D. Kayson E. Davis C.. (2016). Effect of deutetrabenazine on chorea among patients with Huntington disease: a randomized clinical trial. JAMA. 316, 40–50. 10.1001/jama.2016.8655 27380342

[B62] Guo Y. Scarlata S. A. (2013). loss in cellular protein partners promotes alpha-synuclein aggregation in cells resulting from oxidative stress. Biochemistry 52, 3913–3920. 10.1021/bi4002425 23659438 PMC4565189

[B63] Guo Y.-J. Dong S.-Y. Cui X.-X. Feng Y. Liu T. Yin M.. (2016). Resveratrol alleviates MPTP-induced motor impairments and pathological changes by autophagic degradation of alpha-synuclein *via* SIRT1-deacetylated LC3. Mol. Nutr. Food Res. 60, 2161–2175. 10.1002/mnfr.201600111 27296520 PMC6089356

[B64] Haque M. E. Akther M. Jakaria M. Kim I.-S. Azam S. Choi D.-K.. (2020). Targeting the microglial NLRP3 inflammasome and its role in Parkinson's disease. Mov. Disord. 35, 20–33. 10.1002/mds.27874 31680318

[B65] Hervás D. Fornés-Ferrer V. Gómez-Escribano A. P. Sequedo M. D. Peir,ó C. Millán J. M.. (2017). Metformin intake associates with better cognitive function in patients with Huntington's disease. PLoS ONE. 12, e0179283. 10.1371/journal.pone.0179283 28632780 PMC5478119

[B66] Hider R. C. Hoffbrand A. V. (2018). The role of deferiprone in iron chelation. N. Engl. J. Med. 379, 2140–2150. 10.1056/NEJMra1800219 30485781

[B67] Holczer M. Besze B. Zámb,ó V. Csala M. Bánhegyi G. Kapuy O.. (2018). Epigallocatechin-3-gallate (EGCG) promotes autophagy-dependent survival *via* influencing the balance of mTOR-AMPK pathways upon endoplasmic reticulum stress. Oxid. Med. Cell. Longev. 2018, 6721530. 10.1155/2018/6721530 29636854 PMC5831959

[B68] Hou Y. Dan X. Babbar M. Wei Y. Hasselbalch S. G. Croteau D. L.. (2019). Ageing as a risk factor for neurodegenerative disease. Nat. Rev. Neurol. 15, 565–581. 10.1038/s41582-019-0244-7 31501588

[B69] Huang N. Zhang Y. Chen M. Jin H. Nie J. Luo Y.. (2019). Resveratrol delays 6-hydroxydopamine-induced apoptosis by activating the PI3K/Akt signaling pathway. Exp. Gerontol. 124, 110653. 10.1016/j.exger.2019.110653 31295526

[B70] Hung L. W. Villemagne V. L. Cheng L. Sherratt N. A. Ayton S. White A. R.. (2012). The hypoxia imaging agent CuII(atsm) is neuroprotective and improves motor and cognitive functions in multiple animal models of Parkinson's disease. J. Exp. Med. 209, 837–854. 10.1084/jem.20112285 22473957 PMC3328361

[B71] Huntington Study Group Reach2HD Investigators. (2015). Safety, tolerability, and efficacy of PBT2 in Huntington's disease: a phase 2, randomised, double-blind, placebo-controlled trial. Lancet Neurol. 14, 39–47. 10.1016/S1474-4422(14)70262-5 25467848

[B72] Hutton S. R. Otis J. M. Kim E. M. Lamsal Y. Stuber G. D. Snider W. D.. (2017). ERK/MAPK signaling is required for pathway-specific striatal motor functions. J. Neurosci. 37, 8102–8115. 10.1523/JNEUROSCI.0473-17.2017 28733355 PMC5566864

[B73] Jankovic J. Tan E. K. (2020). Parkinson's disease: etiopathogenesis and treatment. J. Neurol. Neurosurg. Psychiatry 91, 795–808. 10.1136/jnnp-2019-322338 32576618

[B74] Javed H. Nagoor Meeran M. F. Azimullah S. Adem A. Sadek B.. (2018). Plant extracts and phytochemicals targeting alpha-synuclein aggregation in Parkinson's disease models. Front. Pharmacol. 9, 1555. 10.3389/fphar.2018.01555 30941047 PMC6433754

[B75] Jeong H. Then F. Jr T. J. M. Mazzulli J. R. Cui L. Savas J. N.. (2009). Acetylation targets mutant huntingtin to autophagosomes for degradation. Cell 137, 60–72. 10.1016/j.cell.2009.03.018 19345187 PMC2940108

[B76] Jimenez-Sanchez M. Licitra F. Underwood B. R. Rubinsztein D. C. (2017). Huntington's disease: mechanisms of pathogenesis and therapeutic strategies. Cold Spring Harb. Perspect. Med. 7, a024240. 10.1101/cshperspect.a024240 27940602 PMC5495055

[B77] Jin J. Gu H. Anders N. M. Ren T. Jiang M. Tao M.. (2016). Metformin protects cells from mutant huntingtin toxicity through activation of AMPK and modulation of mitochondrial dynamics. Neuromolecular Med. 18, 581–592. 10.1007/s12017-016-8412-z 27225841 PMC5112128

[B78] Joppe K. Roser A.-E. Maass F. Lingor P. (2019). The contribution of iron to protein aggregation disorders in the central nervous system. Front. Neurosci. 13, 15. 10.3389/fnins.2019.00015 30723395 PMC6350163

[B79] Junior N. C. F. Dos-Santos-Pereira M. Guimarães F. S. Bel E. D. (2020). Cannabidiol and cannabinoid compounds as potential strategies for treating Parkinson's disease and L-DOPA-induced dyskinesia. Neurotox. Res. 37, 12–29. 10.1007/s12640-019-00109-8 31637586

[B80] Kawabata K. Yoshioka Y. Terao J. (2019). Role of intestinal microbiota in the bioavailability and physiological functions of dietary polyphenols. Molecules 24, 370. 10.3390/molecules24020370 30669635 PMC6359708

[B81] Kelley N. Jeltema D. Duan Y. He Y. (2019). The NLRP3 inflammasome: an overview of mechanisms of activation and regulation. Int. J. Mol. Sci. 20, 3328. 10.3390/ijms20133328 31284572 PMC6651423

[B82] Khan H. Ullah H. Nabavi S. M. (2019). Mechanistic insights of hepatoprotective effects of curcumin: therapeutic updates and future prospects. Food Chem. Toxicol. 124, 182–191. 10.1016/j.fct.2018.12.002 30529260

[B83] Khan M. A. Houck D. R. Gross A. L. Zhang X.-L. Cearley C. Madsen T. M.. (2018). NYX-2925 is a novel NMDA receptor-specific spirocyclic-beta-lactam that modulates synaptic plasticity processes associated with learning and memory. Int. J. Neuropsychopharmacol. 21, 242–254. 10.1093/ijnp/pyx096 29099938 PMC5838819

[B84] Koponen M. Paakinaho A. Lin J. Hartikainen S. Tolppanen A.-M. (2022). Identification of drugs associated with lower risk of parkinson's disease using a systematic screening approach in a nationwide nested case-control study. Clin. Epidemiol. 14, 1217–1227. 10.2147/CLEP.S381289 36325200 PMC9620835

[B85] Kou X. Chen N. (2017). Resveratrol as a natural autophagy regulator for prevention and treatment of Alzheimer's disease. Nutrients 9, 927. 10.3390/nu9090927 28837083 PMC5622687

[B86] Kujawska M. Jodynis-Liebert J. (2018). Polyphenols in Parkinson's disease: a systematic review of *in vivo* studies. Nutrients 10, 642. 10.3390/nu10050642 29783725 PMC5986521

[B87] Kurita K. L. Glassey E. Linington R. G. (2015). Integration of high-content screening and untargeted metabolomics for comprehensive functional annotation of natural product libraries. Proc. Natl. Acad. Sci. USA. 112, 11999–12004. 10.1073/pnas.1507743112 26371303 PMC4593099

[B88] Kusko R. Dreymann J. Ross J. Cha Y. Escalante-Chong R. Garcia-Miralles M.. (2018). Large-scale transcriptomic analysis reveals that pridopidine reverses aberrant gene expression and activates neuroprotective pathways in the YAC128 HD mouse. Mol. Neurodegener. 13, 25. 10.1186/s13024-018-0259-3 29783994 PMC5963017

[B89] Lefèvre-Arbogast S. Gaudout D. Bensalem J. Letenneur L. Dartigues J. F. Hejblum B. P.. (2018). Pattern of polyphenol intake and the long-term risk of dementia in older persons. Neurology 90, e1979–e1988. 10.1212/WNL.0000000000005607 29703769

[B90] Levenson J. M. Schroeter S. Carroll J. C. Cullen V. Asp E. Proschitsky M.. (2016). NPT088 reduces both amyloid-beta and tau pathologies in transgenic mice. Alzheimers. Dement. 2, 141–155. 10.1016/j.trci.2016.06.004 29067301 PMC5651359

[B91] Lévy E. Banna N. E. Baïlle D. Heneman-Masurel A. Truchet S. Rezaei H.. (2019). Causative links between protein aggregation and oxidative stress: a review. Int. J. Mol. Sci. 20, 3896. 10.3390/ijms20163896 31405050 PMC6719959

[B92] Limanaqi F. Biagioni F. Busceti C. L. Ryskalin L. Polzella M. Frati A.. (2019). Phytochemicals bridging autophagy induction and alpha-synuclein degradation in Parkinsonism. Int. J. Mol. Sci. 20, 3274. 10.3390/ijms20133274 31277285 PMC6651086

[B93] Lin K. L. Lin K. J. Wang P. W. Chuang J. H. Lin H. Y. Chen S. D.. (2018). Resveratrol provides neuroprotective effects through modulation of mitochondrial dynamics and ERK1/2 regulated autophagy. Free Radic. Res. 52, 1371–1386. 10.1080/10715762.2018.1489128 30693838

[B94] Liu Q. Zhu D. Jiang P. Tang X. Lang Q. Yu Q.. (2019). Resveratrol synergizes with low doses of L-DOPA to improve MPTP-induced Parkinson disease in mice. Behav. Brain Res. 367, 10–18. 10.1016/j.bbr.2019.03.043 30922940

[B95] Liu W. Wang G. Wang Z. Wang G. Huang J. Liu B.. (2022). Repurposing small-molecule drugs for modulating toxic protein aggregates in neurodegenerative diseases. Drug Discov. Today. 27, 1994–2007. 10.1016/j.drudis.2022.04.003 35395400

[B96] Lv H. Wang Y. Yang X. Ling G. Zhang P. (2022). Application of curcumin nanoformulations in Alzheimer's disease: prevention, diagnosis and treatment. Nutr. Neurosci. 1−16. 10.1080/1028415X.2022.2084550 35694842

[B97] Ma T. C. Buescher J. L. Oatis B. Funk J. A. Nash A. J. Carrier R. L.. (2007). Metformin therapy in a transgenic mouse model of Huntington's disease. Neurosci. Lett. 411, 98–103. 10.1016/j.neulet.2006.10.039 17110029

[B98] Maegawa G. H. B. Tropak M. B. Buttner J. D. Rigat B. A. Fuller M. Pandit D.. (2009). Identification and characterization of ambroxol as an enzyme enhancement agent for Gaucher disease. J. Biol. Chem. 284, 23502–23516. 10.1074/jbc.M109.012393 19578116 PMC2749124

[B99] Maher P. (2019). The potential of flavonoids for the treatment of neurodegenerative diseases. Int. J. Mol. Sci. 20, 3056. 10.3390/ijms20123056 31234550 PMC6627573

[B100] Maher P. Dargusch R. Bodai L. Gerard P. E. Purcell J. M. Marsh J. L. (2011). ERK activation by the polyphenols fisetin and resveratrol provides neuroprotection in multiple models of Huntington's disease. Hum. Mol. Genet. 20, 261–270. 10.1093/hmg/ddq460 20952447 PMC3005900

[B101] Martin-Bastida A. Ward R. J. Newbould R. Piccini P. Sharp D. Kabba C.. (2017). Brain iron chelation by deferiprone in a phase 2 randomised double-blinded placebo controlled clinical trial in Parkinson's disease. Sci. Rep. 7, 1398. 10.1038/s41598-017-01402-2 28469157 PMC5431100

[B102] McColgan P. Tabrizi S. J. (2018). Huntington's disease: a clinical review. Eur. J. Neurol. 25, 24–34. 10.1111/ene.13413 28817209

[B103] McGarry A. Auinger P. Kieburtz K. Geva M. Mehra M. Abler V.. (2020a). Additional safety and exploratory efficacy data at 48 and 60 months from open-hart, an open-label extension study of pridopidine in Huntington disease. J. Huntingtons. Dis. 9, 173–184. 10.3233/JHD-190393 32508327

[B104] McGarry A. Leinonen M. Kieburtz K. Geva M. Olanow C. W. Hayden M.. (2020b). Effects of pridopidine on functional capacity in early-stage participants from the PRIDE-HD study. J. Huntingtons. Dis. 9, 371–380. 10.3233/JHD-200440 33164941 PMC7836066

[B105] McKeage K. Keating G. M. (2011). Fenofibrate: a review of its use in dyslipidaemia. Drugs 71, 1917–1946. 10.2165/11208090-000000000-00000 21942979

[B106] Michelson D. Grundman M. Magnuson K. Fisher R. Levenson J. M. Aisen P.. (2019). Randomized, placebo controlled trial of NPT088, a phage-derived, amyloid-targeted treatment for Alzheimer's disease. J Prev Alzheimers Dis. 6, 228–231. 10.14283/jpad.2019.37 31686093

[B107] Migdalska-Richards A. Daly L. Bezard E. Schapira A. H. V. (2016). Ambroxol effects in glucocerebrosidase and alpha-synuclein transgenic mice. Ann. Neurol. 80, 766–775. 10.1002/ana.24790 27859541 PMC5132106

[B108] Mittal S. Bjørnevik K. Im D. S. Flierl A. Dong X. Locascio J. J.. (2017). beta2-Adrenoreceptor is a regulator of the alpha-synuclein gene driving risk of Parkinson's disease. Science 357, 891–898. 10.1126/science.aaf3934 28860381 PMC5761666

[B109] Mohseni M. Sahebkar A. Askari G. Johnston T. P. Alikiaii B. Bagherniya M.. (2021). The clinical use of curcumin on neurological disorders: an updated systematic review of clinical trials. Phytother. Res. 35, 6862–6882. 10.1002/ptr.7273 34528307

[B110] Morales P. Jagerovic N. (2020). Novel approaches and current challenges with targeting the endocannabinoid system. Expert Opin. Drug Discov. 15, 917–930. 10.1080/17460441.2020.1752178 32336154 PMC7502221

[B111] Mullin S. Smith L. Lee K. D'Souza G. Woodgate P. Elflein J.. (2020). Ambroxol for the treatment of patients with parkinson disease with and without glucocerebrosidase gene mutations: a nonrandomized, noncontrolled trial. JAMA Neurol. 77, 427–434. 10.1001/jamaneurol.2019.4611 31930374 PMC6990847

[B112] Nagle D. G. Ferreira D. Zhou Y. D. (2006). Epigallocatechin-3-gallate (EGCG): chemical and biomedical perspectives. Phytochemistry 67, 1849–1855. 10.1016/j.phytochem.2006.06.020 16876833 PMC2903211

[B113] Naia L. Rosenstock T. R. Oliveira A. M. Oliveira-Sousa S. I. Caldeira G. L. Carmo C.. (2017). Comparative mitochondrial-based protective effects of resveratrol and nicotinamide in Huntington's disease models. Mol. Neurobiol. 54, 5385–5399. 10.1007/s12035-016-0048-3 27590140

[B114] Newman D. J. Cragg G. M. (2020). Natural products as sources of new drugs over the nearly four decades from 01/1981 to 09/2019. J. Nat. Prod. 83, 770–803. 10.1021/acs.jnatprod.9b01285 32162523

[B115] Oertel W. Schulz J. B. (2016). Current and experimental treatments of Parkinson disease: a guide for neuroscientists. *J*. Neurochem. 139, 325–337. 10.1111/jnc.13750 27577098

[B116] Ojha S. Javed H. Azimullah S. Haque M. E. (2016). beta-Caryophyllene, a phytocannabinoid attenuates oxidative stress, neuroinflammation, glial activation, and salvages dopaminergic neurons in a rat model of Parkinson disease. Mol. Cell. Biochem. 418, 59–70. 10.1007/s11010-016-2733-y 27316720

[B117] Oprea T. I. Mestres J. (2012). Drug repurposing: far beyond new targets for old drugs. AAPS J. 14, 759–763. 10.1208/s12248-012-9390-1 22826034 PMC3475856

[B118] Otani K. Shichita T. (2020). Cerebral sterile inflammation in neurodegenerative diseases. Inflamm. Regen. 40, 28. 10.1186/s41232-020-00137-4 33292860 PMC7722432

[B119] Ou Z. Pan J. Tang S. Duan D. Yu D. Nong H.. (2021). Global trends in the incidence, prevalence, and years lived with disability of Parkinson's disease in 204 countries/territories from 1990 to 2019. Front Public Health 9, 776847. 10.3389/fpubh.2021.776847 34950630 PMC8688697

[B120] Paldino E. D'Angelo V. Laurenti D. Angeloni C. Sancesario G. Fusco F. R.. (2020). Modulation of inflammasome and pyroptosis by olaparib, a PARP-1 inhibitor, in the R6/2 mouse model of Huntington's disease. Cells 9, 2286. 10.3390/cells9102286 33066292 PMC7602058

[B121] Parkinson Study Group STEADY-PD III Investigators. (2020). Isradipine versus placebo in early parkinson disease: a randomized trial. Ann. Intern. Med. 172, 591–598. 10.7326/M19-2534 32227247 PMC7465126

[B122] Peball M. Seppi K. Krismer F. Knaus H.-G. Spielberger S. Heim B.. (2022). Effects of nabilone on sleep outcomes in patients with Parkinson's disease: a *post-hoc* analysis of NMS-Nab study. Mov Disord Clin Pract. 9, 751–758. 10.1002/mdc3.13471 35937495 PMC9346252

[B123] Peball M. Werkmann M. Ellmerer P. Stolz R. Valent D. Knaus H.-G.. (2019). Nabilone for non-motor symptoms of Parkinson's disease: a randomized placebo-controlled, double-blind, parallel-group, enriched enrolment randomized withdrawal study (The NMS-Nab Study). J. Neural Transm. 126, 1061–1072. 10.1007/s00702-019-02021-z 31129719 PMC6647387

[B124] Petrussa E. Braidot E. Zancani M. Peresson C. Bertolini A. Patui S.. (2013). Plant flavonoids–biosynthesis, transport and involvement in stress responses. Int. J. Mol. Sci. 14, 14950–14973. 10.3390/ijms140714950 23867610 PMC3742282

[B125] Pinto N. B. Alexandre B. D. Neves K. R. T. Silva A. H. Leal L. K. A. M. Viana G. S. B.. (2015). Neuroprotective properties of the standardized extract from *Camellia sinensis* (Green Tea) and its main bioactive components, epicatechin and epigallocatechin gallate, in the 6-OHDA model of Parkinson's disease. Evid. Based Complement. Alternat. Med. 2015, 161092. 10.1155/2015/161092 26167188 PMC4488543

[B126] Polito C. A. Cai Z.-Y. Shi Y.-L. Li X.-M. Yang R. Shi M.. (2018). Association of tea consumption with risk of Alzheimer's disease and anti-beta-amyloid effects of tea. Nutrients 10, 655. 10.3390/nu10050655 29789466 PMC5986534

[B127] Price D. L. Koike M. A. Khan A. Wrasidlo W. Rockenstein E. Masliah E.. (2018). The small molecule alpha-synuclein misfolding inhibitor, NPT200-11, produces multiple benefits in an animal model of Parkinson's disease. Sci. Rep. 8, 16165. 10.1038/s41598-018-34490-9 30385782 PMC6212487

[B128] Pushpakom S. Iorio F. Eyers P. A. Escott K. J. Hopper S. Wells A.. (2019). Drug repurposing: progress, challenges and recommendations. Nat. Rev. Drug Discov. 18, 41–58. 10.1038/nrd.2018.168 30310233

[B129] Rahman M. H. Akter R. Kamal M. A. (2021). Prospective function of different antioxidant containing natural products in the treatment of neurodegenerative diseases. CNS Neurol. Disord. Drug Targets 20, 694–703. 10.2174/19963181MTA4gNDED1 32703143

[B130] Reale M. Costantini E. Jagarlapoodi S. Khan H. Belwal T. Cichelli A.. (2020). Relationship of wine consumption with Alzheimer's disease. Nutrients 12, 206. 10.3390/nu12010206 31941117 PMC7019227

[B131] Repossi G. Das U. N. Eynard A. R. (2020). Molecular basis of the beneficial actions of resveratrol. Arch. Med. Res. 51, 105–114. 10.1016/j.arcmed.2020.01.010 32111491

[B132] Ringman J. M. Frautschy S. A. Teng E. Begum A. N. Bardens J. Beigi M.. (2012). Oral curcumin for Alzheimer's disease: tolerability and efficacy in a 24-week randomized, double blind, placebo-controlled study. Alzheimers. Res. Ther. 4, 43. 10.1186/alzrt146 23107780 PMC3580400

[B133] Río C. T.-D. Tortajada-Pérez J. Gómez-Escribano A. P. Caster,á F. Peir,ó C. Millán J. M.. (2022). Metformin to treat Huntington disease: a pleiotropic drug against a multi-system disorder. Mech. Ageing Dev. 204, 111670. 10.1016/j.mad.2022.111670 35367225

[B134] Rodrigues F. B. Wild E. J. (2020). Huntington's disease clinical trials corner: April 2020. J. Huntingtons. Dis. 9, 185–197. 10.3233/JHD-200002 32250312

[B135] Rodrigues T. Reker D. Schneider P. Schneider G. (2016). Counting on natural products for drug design. Nat. Chem. 8, 531–541. 10.1038/nchem.2479 27219696

[B136] Roessler H. I. Knoers N. V. A. M. van Haelst M. M. van Haaften G. (2021). Drug repurposing for rare diseases. Trends Pharmacol. Sci. 42, 255–267. 10.1016/j.tips.2021.01.003 33563480

[B137] Rosqvist K. Schrag A. Odin P. Consortium T. C. (2022). Caregiver burden and quality of life in late stage Parkinson's disease. Brain Sci. 12, 111. 10.3390/brainsci12010111 35053854 PMC8773513

[B138] Saeedi M. Eslamifar M. Khezri K. Dizaj S. M. (2019). Applications of nanotechnology in drug delivery to the central nervous system. Biomed. Pharmacother. 111, 666–675. 10.1016/j.biopha.2018.12.133 30611991

[B139] Saeedi M. Eslamifar M. Khezri K. Dizaj S. M. (2022). Drug delivery to the central nervous system. Nat Rev Mater. 7, 314–331. 10.1038/s41578-021-00394-w PMC10923597 38464996

[B140] Saft C. von Hein S. M. Lücke T. Thiels C. Peball M. Djamshidian A.. (2018). Cannabinoids for treatment of dystonia in Huntington's disease. J. Huntingtons. Dis. 7, 167–173. 10.3233/JHD-170283 29562549

[B141] Salehi B. Mishra A. P. Nigam M. Sener B. Kilic M. Sharifi-Rad M.. (2018). Resveratrol: a double-edged sword in health benefits. Biomedicines 6, 91. 10.3390/biomedicines6030091 30205595 PMC6164842

[B142] Sanadgol N. Zahedani S. S. Sharifzadeh M. Khalseh R. Barbari G. R. Abdollahi M.. (2017). Recent updates in imperative natural compounds for healthy brain and nerve function: a systematic review of implications for multiple sclerosis. Curr. Drug Targets 18, 1499–1517. 10.2174/1389450118666161108124414 27829351

[B143] Sanchis A. García-Gimeno M. A. Cañada-Martínez A. J. Sequedo M. D. Millán J. M. Sanz P.. (2019). Metformin treatment reduces motor and neuropsychiatric phenotypes in the zQ175 mouse model of Huntington disease. Exp. Mol. Med. 51, 1–16. 10.1038/s12276-019-0264-9 31165723 PMC6549163

[B144] Sanders M. Chandraratna R. Marek K. Jennings D. (2016). A phase 1 clinical study of the retinoid X receptor (RXR) selective agonist IRX4204 in patients with early Parkinson's disease (PD) (P2, 342.). Neurology. 86.

[B145] Savitt D. Jankovic J. (2019). Targeting alpha-synuclein in Parkinson's disease: progress towards the development of disease-modifying therapeutics. Drugs 79, 797–810. 10.1007/s40265-019-01104-1 30982161

[B146] Schapira A. H. V. Chaudhuri K. R. Jenner P. (2017). Non-motor features of Parkinson disease. Nat. Rev. Neurosci. 18, 435–450. 10.1038/nrn.2017.62 28592904

[B147] Searles Nielsen S. Gross A. Camacho-Soto A. Willis A. W. Racette B. A. (2018). beta2-adrenoreceptor medications and risk of Parkinson disease. Ann. Neurol. 84, 683–693. 10.1002/ana.25341 30225948 PMC6881195

[B148] Silveira C. R. A. MacKinley J. Coleman K. Li Z. Finger E. Bartha R.. (2019). Ambroxol as a novel disease-modifying treatment for Parkinson's disease dementia: protocol for a single-centre, randomized, double-blind, placebo-controlled trial. BMC Neurol. 19, 20. 10.1186/s12883-019-1252-3 30738426 PMC6368728

[B149] Smit J. W. Basile P. Prato M. K. Detalle L. Mathy F.-X. Schmidt A.. (2022). Phase 1/1b studies of UCB0599, an Oral inhibitor of alpha-synuclein misfolding. Including a randomized study in Parkinson's disease. Mov. Disord. 37, 2045–2056. 10.1002/mds.29170 35959805 PMC9804489

[B150] Smith M. R. Syed A. Lukacsovich T. Purcell J. Barbaro B. A. Worthge S. A.. (2014). A potent and selective Sirtuin 1 inhibitor alleviates pathology in multiple animal and cell models of Huntington's disease. Hum. Mol. Genet. 23, 2995–3007. 10.1093/hmg/ddu010 24436303 PMC4031626

[B151] Spathis A. D. Asvos X. Ziavra D. Karampelas T. Topouzis S. Cournia Z.. (2017). Nurr1:RXRalpha heterodimer activation as monotherapy for Parkinson's disease. Proc. Natl. Acad. Sci. USA. 114, 3999–4004. 10.1073/pnas.1616874114 28348207 PMC5393203

[B152] Spencer J. P. (2009). Flavonoids and brain health: multiple effects underpinned by common mechanisms. Genes Nutr. 4, 243–250. 10.1007/s12263-009-0136-3 19685255 PMC2775888

[B153] Süssmuth S. D. Haider S. Landwehrmeyer G. B. Farmer R. Frost C. Tripepi G.. (2015). An exploratory double-blind, randomized clinical trial with selisistat, a SirT1 inhibitor, in patients with Huntington's disease. Br. J. Clin. Pharmacol. 79, 465–476. 10.1111/bcp.12512 25223731 PMC4345957

[B154] Svenningsson P. Johansson A. Nyholm D. Tsitsi P. Hansson F. Sonesson C.. (2018). Safety and tolerability of IRL790 in Parkinson's disease with levodopa-induced dyskinesia-a phase 1b trial. NPJ Parkinsons Dis. 4, 35. 10.1038/s41531-018-0071-3 30534585 PMC6283839

[B155] Tabrizi S. J. Flower M. D. Ross C. A. Wild E. J. (2020). Huntington disease: new insights into molecular pathogenesis and therapeutic opportunities. Nat. Rev. Neurol. 16, 529–546. 10.1038/s41582-020-0389-4 32796930

[B156] Tabrizi S. J. Ghosh R. Leavitt B. R. (2019). Huntingtin lowering strategies for disease modification in Huntington's disease. Neuron 101, 801–819. 10.1016/j.neuron.2019.01.039 30844400

[B157] Teil M. Arotcarena M.-L. Faggiani E. Laferriere F. Bezard E. Dehay B.. (2020). Targeting alpha-synuclein for PD Therapeutics: a pursuit on all fronts. Biomolecules 10, 391. 10.3390/biom10030391 32138193 PMC7175302

[B158] Teleanu D. M. Negut I. Grumezescu V. Grumezescu A. M. Teleanu R. I. (2019). Nanomaterials for drug delivery to the central nervous system. Nanomaterials 9, 371. 10.3390/nano9030371 30841578 PMC6474019

[B159] Terahara N. (2015). Flavonoids in foods: a review. Nat. Prod. Commun. 10, 521–528. 10.1177/1934578X1501000334 25924542

[B160] Trist B. G. Hare D. J. Double K. L. (2019). Oxidative stress in the aging substantia nigra and the etiology of Parkinson's disease. Aging Cell 18, e13031. 10.1111/acel.13031 31432604 PMC6826160

[B161] Troncoso-Escudero P. Sepulveda D. Pérez-Arancibia R. Parra A. V. Arcos J. Grunenwald F.. (2020). On the right track to treat movement disorders: promising therapeutic approaches for Parkinson's and Huntington's DISEASE. Front. Aging Neurosci. 12, 571185. 10.3389/fnagi.2020.571185 33101007 PMC7497570

[B162] Uddin M. S. Mamun A. A. Rahman M. M. Jeandet P. Alexiou A. Behl T.. (2021). Natural products for neurodegeneration: regulating neurotrophic signals. Oxid. Med. Cell. Longev. 2021, 8820406. 10.1155/2021/8820406 34239696 PMC8241508

[B163] Van der Schyf C. J. (2015). Rational drug discovery design approaches for treating Parkinson's disease. Expert Opin. Drug Discov. 10, 713–741. 10.1517/17460441.2015.1041495 26054694

[B164] Varga J. Dér N. P. Zsindely N. Bodai L. (2020). Green tea infusion alleviates neurodegeneration induced by mutant Huntingtin in *Drosophila*. Nutr. Neurosci. 23, 183–189. 10.1080/1028415X.2018.1484021 29973113

[B165] Vauzour D. (2014). Effect of flavonoids on learning, memory and neurocognitive performance: relevance and potential implications for Alzheimer's disease pathophysiology. J. Sci. Food Agric. 94, 1042–1056. 10.1002/jsfa.6473 24338740

[B166] Vauzour D. Vafeiadou K. Rodriguez-Mateos A. Rendeiro C. Spencer J. P. E. (2008). The neuroprotective potential of flavonoids: a multiplicity of effects. Genes Nutr. 3, 115–126. 10.1007/s12263-008-0091-4 18937002 PMC2593006

[B167] Vázquez-Manrique R. P. Farina F. Cambon K. Sequedo M. D. Parker A. J. Millán J. M.. (2016). AMPK activation protects from neuronal dysfunction and vulnerability across nematode, cellular and mouse models of Huntington's disease. Hum. Mol. Genet. 25, 1043–1058. 10.1093/hmg/ddv513 26681807 PMC4764188

[B168] Vidoni C. Secomandi E. Castiglioni A. Melone M. A. B. Isidoro C. (2018). Resveratrol protects neuronal-like cells expressing mutant Huntingtin from dopamine toxicity by rescuing ATG4-mediated autophagosome formation. Neurochem. Int. 117, 174–187. 10.1016/j.neuint.2017.05.013 28532681

[B169] Voulgaropoulou S. D. van Amelsvoort T. A. M. J. Prickaerts J. Vingerhoets C. (2019). The effect of curcumin on cognition in Alzheimer's disease and healthy aging: a systematic review of pre-clinical and clinical studies. Brain Res. 1725, 146476. 10.1016/j.brainres.2019.146476 31560864

[B170] Wan H. Rehngren M. Giordanetto F. Bergström F. Tunek A. (2007). High-throughput screening of drug-brain tissue binding and in silico prediction for assessment of central nervous system drug delivery. J. Med. Chem. 50, 4606–4615. 10.1021/jm070375w 17725338

[B171] Wang J. Bi W. Zhao W. Varghese M. Koch R. J. Walker R. H.. (2016). Selective brain penetrable Nurr1 transactivator for treating Parkinson's disease. Oncotarget 7, 7469–7479. 10.18632/oncotarget.7191 26862735 PMC4884932

[B172] Wang K. Gao Q. Zhang T. Rao J. Ding L. Qiu F.. (2020). Inhibition of CYP2C9 by natural products: insight into the potential risk of herb-drug interactions. Drug Metab. Rev. 52, 35–257. 10.1080/03602532.2020.1758714 32406758

[B173] Wang S. Yuan Y.-H. Chen N.-H. Wang H.-B. (2019). The mechanisms of NLRP3 inflammasome/pyroptosis activation and their role in Parkinson's disease. Int. Immunopharmacol. 67, 458–464. 10.1016/j.intimp.2018.12.019 30594776

[B174] Wang Y. Wu S. Li Q. Lang W. Li W. Jiang X.. (2022). Epigallocatechin-3-gallate: a phytochemical as a promising drug candidate for the treatment of Parkinson's disease. Front. Pharmacol. 13, 977521. 10.3389/fphar.2022.977521 36172194 PMC9511047

[B175] Waters S. Sonesson C. Svensson P. Tedroff J. Carta M. Ljung E.. (2020). Preclinical pharmacology of [2-(3-fluoro-5-methanesulfonylphenoxy)ethyl](propyl)amine (IRL790), a novel dopamine transmission modulator for the treatment of motor and psychiatric complications in Parkinson's disease. J. Pharmacol. Exp. Ther. 374, 113–125. 10.1124/jpet.119.264226 32358046

[B176] Waters S. Tedroff J. Ponten H. Klamer D. Sonesson C. Waters N.. (2018). Pridopidine: overview of pharmacology and rationale for its use in Huntington's disease. J. Huntingtons. Dis. 7, 1–16. 10.3233/JHD-170267 29480206 PMC5836399

[B177] Wrasidlo W. Tsigelny I. F. Price D. L. Dutta G. Rockenstein E. Schwarz T. C.. (2016). A *de novo* compound targeting alpha-synuclein improves deficits in models of Parkinson's disease. Brain 139(Pt 12), 3217–36. 10.1093/brain/aww238 27679481 PMC5840882

[B178] Xu Y. Zhang Y. Quan Z. Wong W. Guo J. Zhang R.. (2016). Epigallocatechin gallate (EGCG) inhibits alpha-synuclein aggregation: a potential agent for Parkinson's disease. Neurochem. Res. 41, 2788–2796. 10.1007/s11064-016-1995-9 27364962

[B179] Yero T. Rey J. A. (2008). Tetrabenazine (xenazine), an FDA-approved treatment option for Huntington's disease-related chorea. P T. 33, 690–694. 19750050 PMC2730806

[B180] Zhang H. Bai L. He J. Zhong L. Duan X. Ouyang L.. (2017). Recent advances in discovery and development of natural products as source for anti-Parkinson's disease lead compounds. Eur. J. Med. Chem. 141, 257–272. 10.1016/j.ejmech.2017.09.068 29031072

[B181] Zhang L.-F. Yu X.-L. Ji M. Liu S.-Y. Wu X.-L. Wang Y.-J.. (2018). Resveratrol alleviates motor and cognitive deficits and neuropathology in the A53T alpha-synuclein mouse model of Parkinson's disease. Food Funct. 9, 6414–6426. 10.1039/C8FO00964C 30462117

[B182] Zhou H. Beevers C. S. Huang S. (2011). The targets of curcumin. Curr. Drug Targets 12, 332–347. 10.2174/138945011794815356 20955148 PMC3025067

